# Long COVID is not the same for everyone: a hierarchical cluster analysis of Long COVID symptoms 9 and 12 months after SARS-CoV-2 test

**DOI:** 10.1186/s12879-024-09896-8

**Published:** 2024-09-19

**Authors:** Marta Moniz, Carolina Ruivinho, Ana Rita Goes, Patrícia Soares, Andreia Leite

**Affiliations:** 1 Public Health Research Centre, Comprehensive Health Research Center, NOVA National School of Public Health, CHRC, REAL, CCAL, NOVA University Lisbon, Lisbon, Portugal; 2 National Institute of Health Doutor Ricardo Jorge, Lisbon, Portugal

**Keywords:** Symptoms cluster, Long COVID, Portugal, Public health

## Abstract

**Background:**

Identifying symptom clusters in Long COVID is necessary for developing effective therapies for this diverse condition and improving the quality of life of those affected by this heterogeneous condition. In this study, we aimed to identify and compare symptom clusters at 9 and 12 months after a SARS-CoV-2 positive test and describe each cluster regarding factors at infection.

**Methods:**

This is a cross-sectional study with individuals randomly selected from the Portuguese National System of Epidemiological Surveillance (SINAVE) database. Individuals who had a positive SARS-CoV-2 test in August 2022 were contacted to participate in a telephonic interview approximately 9 and 12 months after the test. A hierarchical clustering analysis was performed, using Euclidean distance and Ward’s linkage. Clustering was performed in the 35 symptoms reported 9 and 12 months after the SARS-CoV-2 positive test and characterised considering age, sex, pre-existing health conditions and symptoms at time of SARS-CoV-2 infection.

**Results:**

552 individuals were included at 9 months and 458 at 12 months. The median age was 52 years (IQR: 40–64 years) and 59% were female. Hypertension and high cholesterol were the most frequently reported pre-existing health conditions. Memory loss, fatigue or weakness and joint pain were the most frequent symptoms reported 9 and 12 months after the positive test. Four clusters were identified at both times: no or minor symptoms; multi-symptoms; joint pain; and neurocognitive-related symptoms. Clusters remained similar in both times, but, within the neurocognitive cluster, memory loss and concentration issues increased in frequency at 12 months. Multi-symptoms cluster had older people, more females and more pre-existing health conditions at 9 months. However, at 12 months, older people and those with more pre-existing health conditions were in joint pain cluster.

**Conclusions:**

Our results suggest that Long COVID is not the same for everyone. In our study, clusters remained similar at 9 and 12 months, except for a slight variation in the frequency of symptoms that composed each cluster. Understanding Long COVID clusters might help identify treatments for this condition. However, further validation of the observed clusters and analysis of its risk factors is needed.

**Supplementary Information:**

The online version contains supplementary material available at 10.1186/s12879-024-09896-8.

## Introduction

The outbreak caused by the severe acute respiratory syndrome coronavirus 2 (SARS-CoV-2), which resulted in COVID-19 disease, has rapidly evolved into a global pandemic [1]. While the infection acute phase has been extensively documented, emerging evidence suggests that a substantial proportion of individuals experience long-term symptoms that are not yet fully understood. These are often referred to as “Long COVID” or “post-COVID condition (PCC)” [2], and can significantly impact the quality of life and functional capacity of affected individuals [3], posing significant challenges to public health worldwide.

Different individual characteristics associated with different symptomatology and contradictory evidence regarding the risk factors for Long COVID symptoms [4–6] indicate that Long COVID is not a uniform syndrome. Previous studies have assessed potential Long COVID symptom clusters. A systematic review of such studies showed that neurological clustering was consistently identified, followed by cardiorespiratory and systemic/inflammatory [7]. Yet only eight studies were included, and the number and characteristics of clusters identified varied widely. Kenny et al. identified musculoskeletal and pain-related symptoms, cardiorespiratory symptoms and lower number of reported symptoms [8]; Nayani et al. identified loss of smell and taste, neurological symptoms and multiple symptoms classes [9]; Gentilotti et al. identified chronic fatigue-like syndrome, respiratory syndrome, chronic pain syndrome and neurosensorial syndrome [10]; Goldhaber et al. identified gastrointestinal, musculoskeletal, neurocognitive, airway and cardiopulmonary clusters [11]; Torrel et al. identified multisystemic, multisystemic – predominantly dysautonomous, heterogeneous, taste & smell, and menstrual & sexual alterations [12]; van den Houdt et al. identified moderate inflammatory symptoms, high inflammatory symptoms, moderate malaise-neurocognitive symptoms, high malaise-neurocognitive-psychosocial symptoms, low overall symptoms, and high overall symptoms [13]. This might be due to the variability in symptom selection [11, 12], symptom collection [8, 9], and clustering methodology [8–12]. Studies analysing symptom clusters focused either on the early stages of post-infection symptoms (> 3 months [9, 12, 14] or later stages (≥ 12 months [10, 15–17]), and there is little evidence regarding symptoms cluster between these two periods. Thus, clustering of Long COVID symptoms requires further assessment.

Understanding Long COVID has been a complex challenge, indicating that its treatment is more likely to be paved with tailored interventions rather than a one-size-fits-all approach [18]. This knowledge may facilitate the design of tailored interventions and ultimately improve the overall quality of life for individuals affected by this condition [19]. Hence, this study aimed to identify and compare symptom clusters at 9 and 12 months after a SARS-CoV-2 positive test and describe each cluster regarding factors at infection.

## Methods

### Data collection and study population

This study included individuals who had a SARS-CoV-2 test notification from the National System of Epidemiological Surveillance (SINAVE) in August 2022 and lived in Lisbon and Tagus Valley, which comprises one-third of the country's population distributed between urban and rural, high and low population density areas. Individuals who had a positive SARS-CoV-2 test in August 2022 (both PCR and rapid antigen test), were residing in Lisbon and Tagus Valley region during the study period regardless of their nationality or immigration status, were 18 years old or older, and consented to participate were included in the study. Individuals without a valid landline or mobile phone registered; institutionalised (e.g. residential structures for the elderly, prisons); who died between the date of the test and the call; with language difficulties (languages not covered by the group of translators that were part of the team of investigators) or deafness, as well as advanced states of mental illness or dementia; who were emigrants on holidays in Portugal or Portuguese tourists; and who tested positive for SARS-CoV-2 after August 2022 but before completing the first questionnaire and before the second questionnaire, to ensure an homogenous time between the test and symptoms were excluded.

The General Directorate of Health (DGS) provided information on a list of potential participants in two phases: 1) names and contact numbers (landline or mobile) of a random sample of 10,000 individuals who had a SARS-CoV-2 test approximately 9 months before the beginning of data collection. Verbal informed consent was obtained over the telephone by a trained inquirer; 2) result of the test for SARS-CoV-2. The second phase was only applicable to the individuals who consented to participate in the study. A questionnaire was performed between June 12 and August 8 of 2023, through a 30-minute computer-assisted telephone interview. Calls were scheduled for the most convenient times for the participants, with a maximum of five call attempts at different hours. Information collected included individuals’ sociodemographic information, previous health conditions, previous lifestyle behaviours (alcohol intake and smoking) and symptoms reported at the time of the SARS-CoV-2 test and during the interview. Three months after the first questionnaire, a second similar questionnaire was performed on those who accepted to continue in the study after the first questionnaire. This second questionnaire only questioned the participant regarding symptoms felt and changes in health conditions 12 months after the test to assess the evolution of the participants health between the two time points. Further information on the data collection process and study population can be found in the published study protocol [20] and both questionnaires can be seen in Additional file 1.

### Variables

We considered the presence of at least one symptom reported 9 and 12 months after the SARS-CoV-2 test for the clustering analysis. The symptoms considered included persistent or worsening of usual cough, difficulty breathing, runny nose, sore throat, chest pain, abdominal pain, vomiting or nausea, diarrhoea, fever ≥ 38°C, chills, headache, joint pain, myalgia, change or loss of smell, change or loss of taste, fatigue or weakness, breathing pain, palpitations, loss of appetite, constipation, difficulties urinating, swollen ankle, balance issues, not feeling one side of the body or face, tingling, fainting, seizures, tremors, swallowing difficulties, chewing difficulties, tinnitus, insomnia, rash, concentration issues and memory loss. The symptoms were based on the International Severe Acute Respiratory and Emerging Infection Consortium (ISARIC)/WHO COVID-19 clinical characterisation protocol [20] and derived from the question “I would like you to tell me whether or not if, in the last 7 days, you have felt any or some of them, which you had not felt before the test was taken, 9 (and 12) months ago, in August 2022”. Due to cluster analysis requirements, individuals with missing values were removed from the analysis.

Sex (male/female), age (in years), behavioural and clinical characteristics: smoking (yes/no), alcohol intake (never/ 2 to 4 times a month or less/ twice a week or more), overweight (yes/no) and pre-existing health conditions (COVID-19, hypertension, diabetes, high cholesterol, asthma, chronic bronchitis, pulmonary fibrosis, heart failure, reflux disease, psychiatric disease, myocardial infarction, stroke, deep vein thrombosis, pulmonary thromboembolism) and symptoms reported at the time of SARS-CoV-2 test (persistent cough or worsening of usual cough, difficulty breathing, runny nose, sore throat, chest pain, abdominal pain, vomiting or nausea, diarrhoea, fever ≥ 38°C, chills, headache, joint pain, myalgia, change or loss of smell, change or loss of taste, fatigue or weakness) were also used to characterise individuals within each cluster. Body mass index equal or above 25.0 Kg/m^2^ was considered overweight, based on the WHO criteria [21].

### Statistical analysis

Categorical data was summarised as frequencies and percentages, and continuous data as mean (minimum and maximum) and median (alongside the corresponding interquartile range (IQR) presented as the 25th and 75th percentile).

A hierarchical clustering analysis was performed to identify clusters within the 35 symptoms reported 9 months after SARS-CoV-2 diagnosis. This method starts with each participant as its own cluster, combining the most “similar” participants based on closeness (Euclidean distance), continuing until the last two clusters merge into one cluster containing all participants. Wards linkage was used to assess the distance between each cluster. Hierarchical clustering works especially well with smaller datasets since it does not require the number of clusters to be specified in advance like other clustering methods. The optimal number of clusters was chosen based on visual inspection (dendrogram), symptoms included in each cluster (i.e. having symptoms in a cluster that were relatable between them), silhouette score and Dunn’s index. The same methodology was also applied to the symptoms reported at 12 months to compare clusters. Data were analysed using R version 4.0.3 [22] and the hierarchical clustering was performed with *cluster* package (2.1.6) [23].

## Results

### Individuals’ characteristics

We included 552 SARS-CoV-2 positive individuals who completed the questionnaire at 9 months and 424 at 12 months. At 9 months, the median age was 52 years (IQR: 40–64 years) and 59% were female. Considering characteristics of individuals before the test, most individuals did not smoke (79%) and were overweight (55%), but only 26% drank alcohol two times a week or more. Hypertension (30%) and high cholesterol (29%) were the most frequently reported diagnosed pre-existing health condition. Conversely, the less frequent pre-existing health conditions reported were pulmonary fibrosis (0.4%) and pulmonary thrombosis (0.5%). Considering the symptoms reported at the time of the test, runny nose, sore throat, headache, myalgia, fatigue or weakness were the ones with higher frequency. The distribution of characteristics before and at the time of the test for the individuals included at 12 months remained similar. Sociodemographic and clinical features of the study population can be found in Additional file 2.

**Fig. 1:**
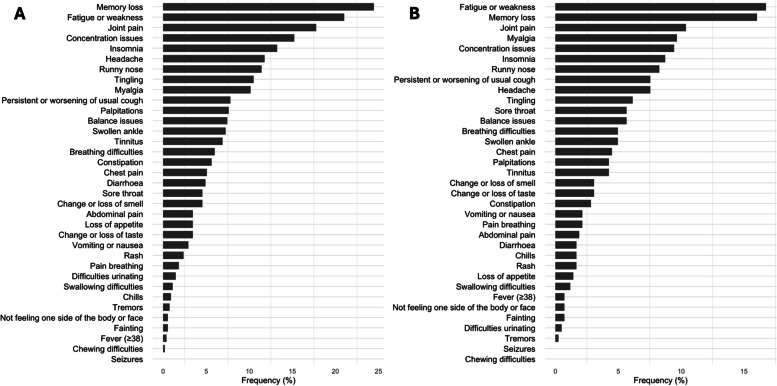
Frequency of Long COVID symptoms 9 (**A**) and 12 (**B**) months after a SARS-CoV-2 positive test

Figure 1 shows the symptoms reported 9 (panel A), and 12 months (panel B) after the positive SARS-CoV-2 test. Memory loss, fatigue or weakness and joint pain were the most frequent symptoms at both times. However, all symptoms are reduced in frequency in the second moment.

### Clustering analysis

**Fig. 2:**
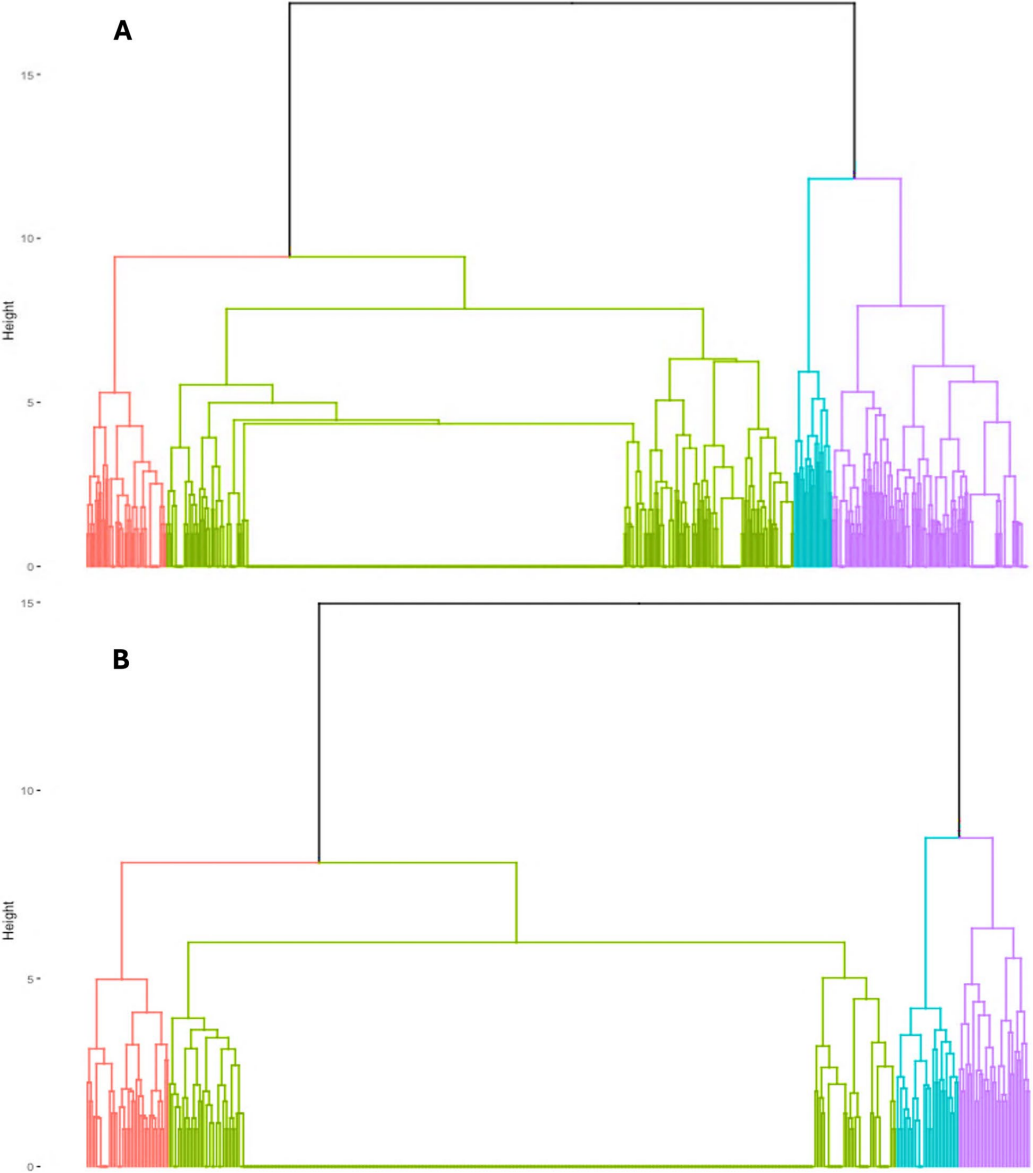
Dendrogram based on hierarchical clustering analysis of 37 symptoms reported (**A**) 9 months after SARS-CoV-2 positive test, and (**B**) 12 months after SARS-CoV-2 positive test

Based on the dendrograms (Fig. 2), we considered two options: three and four clusters. Statistics for both options yielded similar metrics, although the option with four clusters was chosen because it was the most reasonable considering both statistic tests and cluster’s explanation. Validation statistics for both options and the symptom distribution considering the option with three clusters are provided in Additional file 3, in Table 1 and Fig. 1, respectively.

**Fig. 3:**
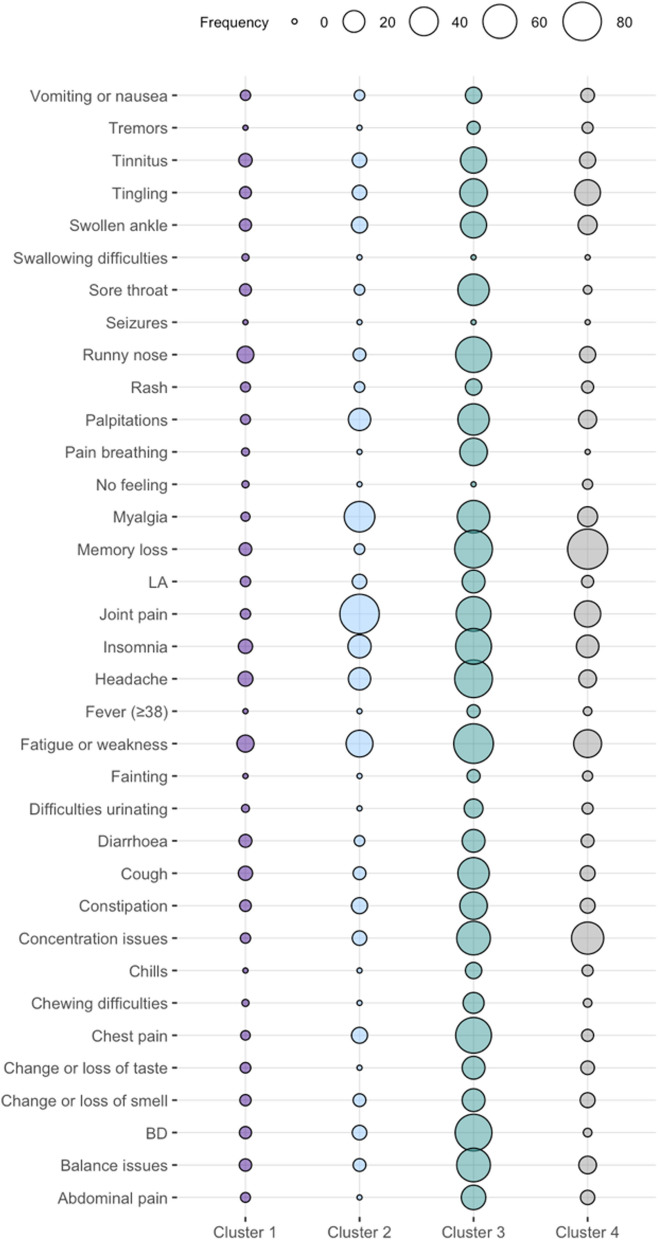
Bubble plot of symptom cluster frequency derived from hierarchical clustering of Long COVID symptoms 9 months after a SARS-COV-2 positive test

**Fig. 4:**
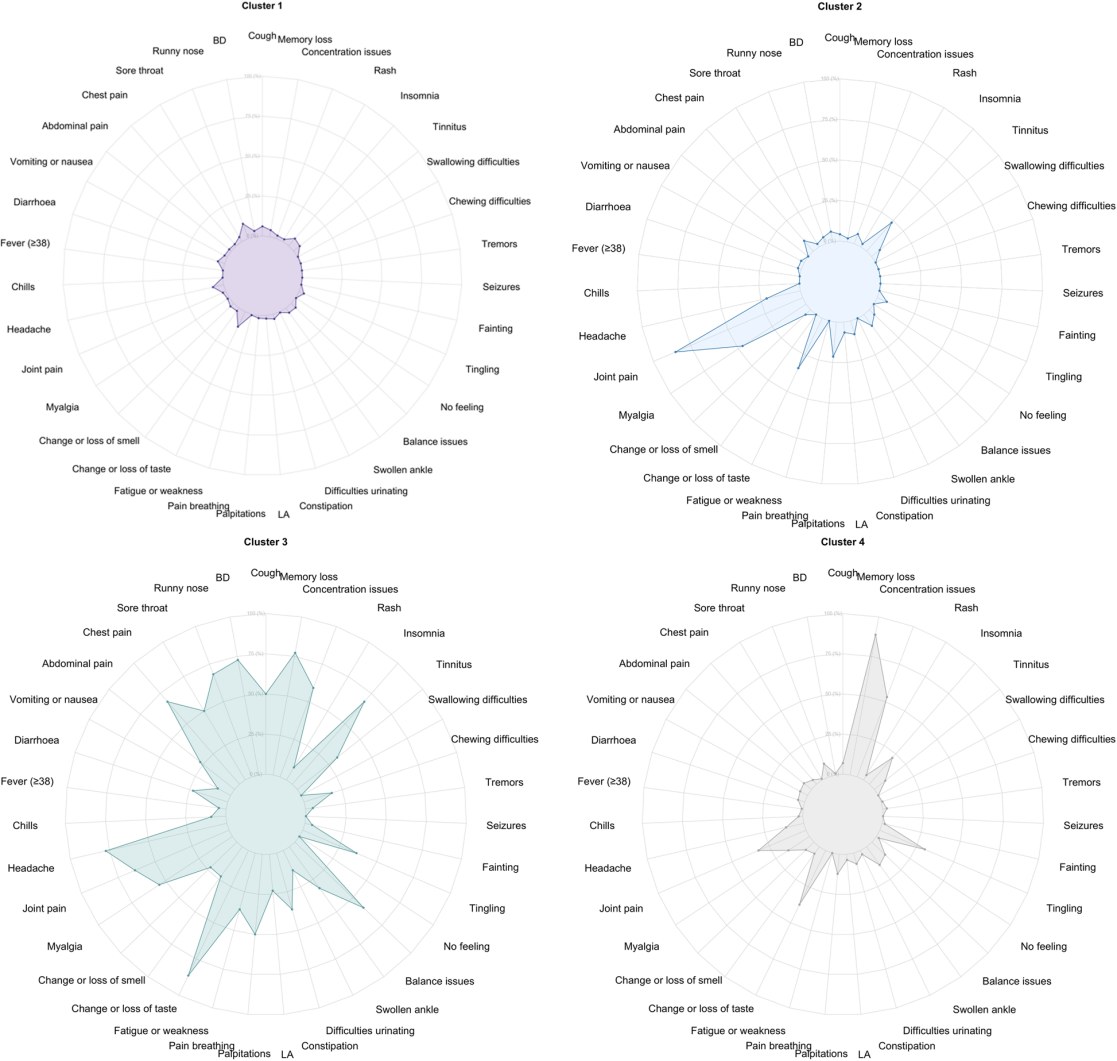
Radar plots displaying the frequency of Long COVID symptoms across all four clusters. Abbreviations: LA: Loss of appetite; No feeling: Not feeling one side of the body or face; BD: Breathing difficulties

**Fig. 5:**
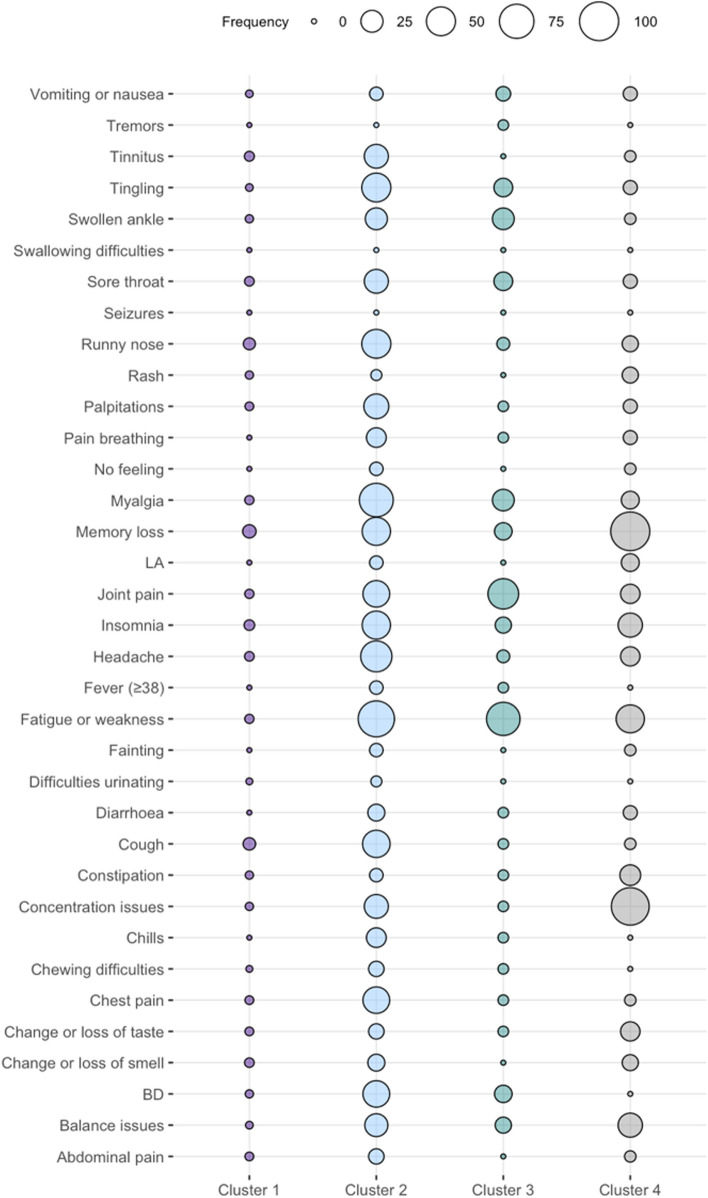
Bubble plot of symptom cluster frequency derived from hierarchical clustering of Long COVID symptoms 12 months after a SARS-COV-2 positive test

**Fig. 6:**
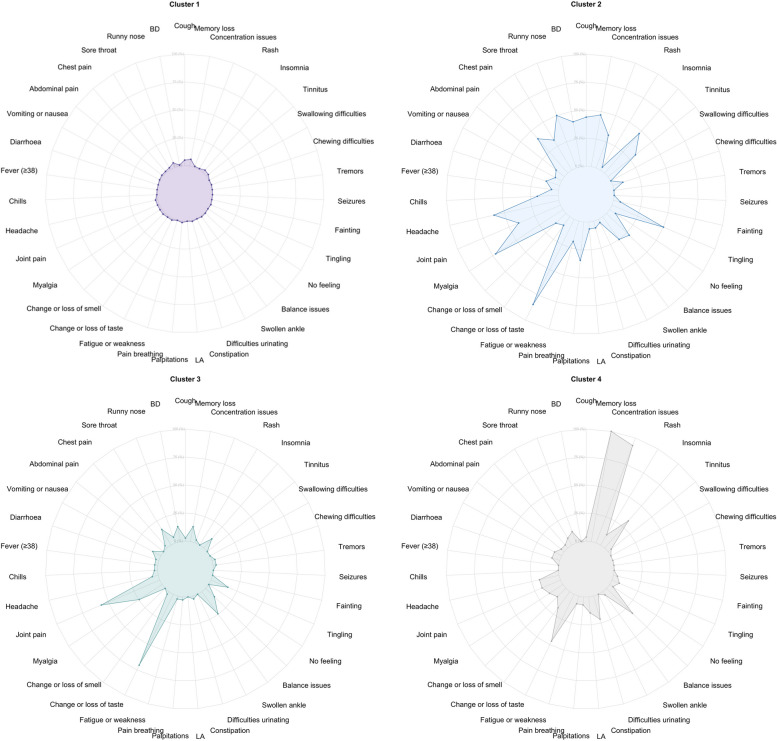
Radar plots displaying the frequency of Long COVID symptoms across all four clusters. Abbreviations: LA: Loss of appetite; No feeling: Not feeling one side of the body or face; BD: Breathing difficulties

Figures 3 and 4 show the distribution of the symptoms across the four clusters at 9 months. Cluster 1 (*n* = 368, 67%) was characterised by individuals with no or minor symptoms, and the most frequent symptoms were runny nose (10%) and fatigue (10%). Cluster 2 (*n* = 47, 8%) was characterised by individuals with a higher frequency of joint (85%) and myalgia (47%). Cluster 3 (*n* = 22, 4%) included individuals with multiple symptoms across different organs, and the most frequent symptoms were fatigue (86%), memory loss (77%) and headache (77%). Cluster 4 (*n* = 115, 21%) was characterised by individuals with cognitive-related symptoms, and the most frequent symptoms were memory loss (89%) and concentration issues (53%). Figures 5 and 6 show the distribution of the symptoms across the four clusters at 12 months, which remained similar to the clusters identified at 9 months but with some differences regarding symptoms distribution. Cluster 1 (*n* = 327, 71%) included individuals with no or minor symptoms, and the most prevalent symptoms were memory loss (6%), cough (5%) and runny nose (4%). Cluster 2 (*n* = 32, 7%) included individuals with multiple symptoms across different organs, in which fatigue (84%) and myalgia (72%) were the most prevalent symptoms. Cluster 3 (*n* = 37, 8%) was characterised by the individuals who reported mainly joint pain (57%) and fatigue (70%). Cluster 4 (*n* = 28, 6%) included individuals with cognitive-related symptoms in which concentration issues (93%) was the most prevalent symptom, and all of them reported memory loss (100%).

The cluster characterised by minor or no symptoms remained the most frequent and the cluster characterised by cognitive-related symptoms went from the second most frequent to the least frequent. Overall, symptom’s frequency fluctuated between the two time points. The cluster characterised by no or minor symptoms, had runny nose and fatigue as the most frequent symptoms at 9 months, while memory loss and cough were more common at 12 months. The cluster characterised by joint pain, showed myalgia as the second most frequent symptom at 9 months, with fatigue becoming more frequent at 12 months. In the multi-symptoms cluster, individuals reported fatigue most frequently at both time points. However, memory loss and headache were the second most frequent symptoms at 9 months, and myalgia at 12 months. In the neurocognitive-related symptoms cluster, memory loss and concentration issues were prevalent at both time points. Although the order of clusters 2 and 3 have changed between the 9 and 12 months analysis, possibly due to the different data used, the clusters identified remained consistent over time.

### Characteristics of the individuals per clusters

The characteristics of individuals in each symptom cluster at 9 months after SARS-CoV-2 test are shown in Table 1. Cluster 1, characterised by no or minor symptoms, had the highest median age (62 years) and the highest percentage of females (90%). Females were predominant in all clusters. Cluster 3 had the highest percentage of pre-existing health conditions. Fatigue and headache were consistently among the most frequent symptoms at testing in all clusters. In Cluster 3, these symptoms were particularly prevalent, with fatigue affecting 100% of individuals and headache affecting 86%. Hypertension and high cholesterol were the pre-existing health conditions most frequent in all clusters, and cluster 3 had the highest frequency of pre-existing health conditions.

**Table 1:** Participants’ sociodemographic and clinical characteristics by clusters 9 months after the positive SARS-CoV-2 test (*N* = 552)

	Clusters			
**Variables**	1 (*n* = 368)	2 (*n* = 47)	3 (*n* = 22)	4 (*n* = 115)
**Age**				
Mean (Range)	51.93 (19.00, 92.00)	53.21 (25.00, 86.00)	59.77 (22.00, 82.00)	53.22 (18.00, 87.00)
Median (IQR)	52.00 (39.75, 64.00)	52.00 (44.50, 61.00)	62.00 (50.50, 67.00)	52.00 (40.50, 64.50)
**Sex (*n*** **= 546)**				
Female	188 (51.51%)	32 (68.09%)	19 (90.48%)	81 (71.68%)
Male	177 (48.49%)	15 (31.91%)	2 (9.52%)	32 (28.32%)
Tobacco consumptiona (*n* = 551)	76 (20.65%)	12 (26.09%)	5 (22.73%)	23 (20.00%)
**Alcohol consumption**				
Never	107 (29.08%)	14 (29.79%)	8 (36.36%)	24 (20.87%)
2 to 4 times a month or less	162 (44.02%)	26 (55.32%)	8 (36.36%)	62 (53.91%)
2 times a week or more	99 (26.90%)	7 (14.89%)	6 (27.27%)	29 (25.22%)
Overweight^a^ (*n* = 544)	198 (54.25%)	28 (63.64%)	14 (63.64%)	61 (53.98%)
**Pre-existing health conditions**				
COVID-19^a^ (*n* = 543)	74 (20.44%)	12 (26.09%)	6 (27.27%)	29 (25.66%)
Hypertension^a^ (*n* = 550)	104 (28.26%)	14 (30.43%)	9 (40.91%)	36 (31.58%)
Diabetes^a^ (*n* = 549)	39 (10.63%)	3 (6.52%)	1 (4.55%)	10 (8.77%)
High cholesterol^a^ (*n* = 549)	106 (28.80%)	7 (15.56%)	7 (31.82%)	38 (33.33%)
Asthma^a^ (*n* = 550)	31 (8.42%)	5 (10.87%)	2 (9.09%)	11 (9.65%)
Bronchitis^a^ (*n* = 550)	13 (3.53%)	2 (4.35%)	3 (13.64%)	5 (4.39%)
Pulmonary fibrosis^a^ (*n* = 549)	0 (0.00%)	1 (2.17%)	1 (4.55%)	0 (0.00%)
Heart failure^a^ (*n* = 550)	16 (4.35%)	1 (2.17%)	6 (27.27%)	4 (3.51%)
Reflux disease^a^ (*n* = 550)	33 (8.97%)	5 (10.87%)	6 (27.27%)	17 (14.91%)
Psychiatric condition^a^ (*n* = 549)	49 (13.35%)	11 (23.91%)	6 (27.27%)	27 (23.68%)
Myocardial infarction^a^ (*n* = 548)	11 (3.01%)	2 (4.35%)	1 (4.55%)	0 (0.00%)
Stroke^a^ (*n* = 548)	9 (2.46%)	1 (2.17%)	1 (4.55%)	0 (0.00%)
Thrombosis^a^ (*n* = 548)	5 (1.37%)	0 (0.00%)	2 (9.09%)	1 (0.88%)
Pulmonary thrombosis^a^ (*n* = 549)	1 (0.27%)	0 (0.00%)	1 (4.55%)	1 (0.88%)
Pre-existing health condition^a^	264 (71.74%)	33 (70.21%)	18 (81.82%)	87 (75.65%)
**Symptoms reported at the time of SARS-CoV-2 test**				
Cough^a^ (*n* = 546**)**	153 (41.80%)	21 (46.67%)	13 (59.09%)	54 (47.79%)
Breathing difficulties^a^ (*n* = 549)	56 (15.26%)	8 (17.39%)	15 (68.18%)	22 (19.30%)
Runny nose^a^ (*n* = 541)	181 (50.00%)	27 (58.70%)	14 (66.67%)	64 (57.14%)
Sore throat^a^ (*n* = 544)	180 (49.18%)	26 (55.32%)	11 (57.89%)	64 (57.14%)
Chest pain^a^ (*n* = 543)	28 (7.67%)	7 (15.22%)	7 (33.33%)	12 (10.81%)
Abdominal pain^a^ (*n* = 546)	22 (6.01%)	2 (4.44%)	4 (18.18%)	13 (11.50%)
Vomiting or nause^a^ (*n* = 548)	34 (9.29%)	6 (13.04%)	7 (31.82%)	21 (18.42%)
Diarrhoea^a^ (*n* = 543)	39 (10.74%)	5 (10.87%)	3 (14.29%)	19 (16.81%)
Fever (≥ 38°C)^a^ (*n* = 539)	161 (44.85%)	22 (48.89%)	9 (42.86%)	54 (47.37%)
Chills^a^ (*n* = 540)	143 (39.83%)	23 (50.00%)	13 (61.90%)	70 (61.40%)
Headache^a^ (*n* = 549)	196 (53.41%)	31 (65.96%)	19 (86.36%)	73 (64.60%)
Joint pain^a^ (*n* = 542)	140 (38.57%)	32 (69.57%)	18 (85.71%)	58 (51.79%)
Myalgia^a^ (*n* = 547)	179 (49.18%)	35 (74.47%)	18 (81.82%)	70 (61.40%)
Change or loss of smell^a^ (*n* = 545)	72 (19.67%)	12 (27.27%)	11 (50.00%)	37 (32.74%)
Change or loss of taste^a^ (*n* = 547)	72 (19.67%)	14 (30.43%)	9 (40.91%)	29 (25.66%)
Fatigue or weakness^a^ (*n* = 548)	182 (49.86%)	33 (70.21%)	22 (100.00%)	91 (79.82%)
Any symptom^a^	337 (91.58%)	45 (95.74%)	22 (100.00%)	111 (96.52%)

^a^the category represented is “yes”

Table 2 shows the characteristics of individuals in each symptom cluster at 12 months after SARS-CoV-2 test. Table 2 shows the characteristics of individuals in each symptom cluster at 12 months after SARS-CoV-2 test. Cluster 2, characterised by multiple symptoms, included the highest percentage of females (84%). Cluster 3 (joint pain) displayed the highest median age (58 years) and the highest frequency of tobacco consumption (30%). Cluster 4 presented the highest frequency of alcohol consumption (36%). Females were also predominant in all clusters at 12 months, and the frequency of pre-existing health conditions was similar between clusters. Fatigue was the most frequent symptom at testing in all clusters, having the highest frequency in cluster 2, with 88%.

**Table 2:** Participants’ sociodemographic and clinical characteristics by clusters 12 months after the positive SARS-CoV-2 test (*N* = 424)

	Clusters			
**Variables**	**1** (*n* = 327)	**2** (*n* = 32)	**3** (*n* = 37)	**4** (*n* = 28)
**Age**				
Mean (Range)	52.60 (19.00, 92.00)	55.69 (26.00, 80.00)	55.73 (22.00, 89.00)	53.75 (18.00, 87.00)
Median (IQR)	51.00 (40.00, 65.50)	53.50 (43.75, 69.00)	58.00 (50.00, 62.00)	53.00 (39.50, 65.00)
Sex (*n* = 418)				
Female	175 (54.01%)	26 (83.87%)	26 (72.22%)	20 (74.07%)
Male	149 (45.99%)	5 (16.13%)	10 (27.78%)	7 (25.93%)
Tobacco consumption^a^	63 (19.27%)	6 (18.75%)	11 (29.73%)	4 (14.29%)
**Alcohol consumption**				
Never	94 (28.75%)	12 (37.50%)	9 (24.32%)	8 (28.57%)
2 to 4 times a month or less	154 (47.09%)	13 (40.63%)	18 (48.65%)	10 (35.71%)
2 times a week or more	79 (24.16%)	7 (21.88%)	10 (27.03%)	10 (35.71%)
** Overweight**^**a**^ **(*n*** **= 418)**	173 (53.73%)	21 (65.63%)	22 (61.11%)	16 (57.14%)
**Pre-existing health conditions**				
COVID-19^a^ (*n* = 415)	63 (19.63%)	14 (46.67%)	8 (22.22%)	6 (21.43%)
Hypertension^a^ (*n* = 422)	101 (30.89%)	12 (38.71%)	10 (27.78%)	9 (32.14%)
Diabetes^a^ (*n* = 421)	33 (10.09%)	4 (13.33%)	5 (13.89%)	4 (14.29%)
High cholesterol^a^ (*n* = 422)	100 (30.58%)	14 (45.16%)	7 (19.44%)	13 (46.43%)
Asthma^a^ (*n* = 422)	27 (8.26%)	7 (22.58%)	4 (11.11%)	3 (10.71%)
Bronchitis^a^ (*n* = 421)	9 (2.75%)	4 (12.90%)	3 (8.33%)	1 (3.57%)
Pulmonary fibrosis^a^ (*n* = 421)	1 (0.31%)	1 (3.23%)	0 (0.00%)	0 (0.00%)
Heart failure^a^ (*n* = 422)	13 (3.98%)	2 (6.45%)	2 (5.56%)	2 (7.14%)
Reflux disease^a^ (*n* = 422)	30 (9.17%)	6 (19.35%)	5 (13.89%)	4 (14.29%)
Psychiatric condition^a^ (*n* = 421)	52 (15.95%)	10 (32.26%)	9 (25.00%)	9 (32.14%)
Myocardial infarction^a^ (*n* = 421)	8 (2.45%)	0 (0.00%)	1 (2.78%)	1 (3.57%)
Stroke^a^ (*n* = 421)	6 (1.84%)	1 (3.23%)	2 (5.56%)	1 (3.57%)
Thrombosis^a^ (*n* = 420)	4 (1.23%)	0 (0.00%)	0 (0.00%)	0 (0.00%)
Pulmonary thrombosis^a^ (*n* = 419)	1 (0.31%)	0 (0.00%)	0 (0.00%)	0 (0.00%)
Any pre-existing health condition^a^	241 (73.70%)	29 (90.63%)	28 (75.68%)	21 (75.00%)
**Symptoms reported at the time of SARS-CoV-2 test**				
Cough^a^ (*n* = 419)	144 (44.31%)	17 (53.13%)	18 (50.00%)	12 (46.15%)
Breathing difficulties^a^ (*n* = 423)	53 (16.21%)	15 (46.88%)	7 (19.44%)	6 (21.43%)
Runny nose^a^ (*n* = 414)	175 (55.03%)	16 (51.61%)	21 (56.76%)	16 (57.14%)
Sore throat^a^ (*n* = 419)	169 (52.32%)	18 (58.06%)	23 (62.16%)	12 (42.86%)
Chest pain^a^ (*n* = 419)	23 (7.10%)	6 (18.75%)	3 (8.33%)	4 (14.81%)
Abdominal pain^a^ (*n* = 421)	25 (7.69%)	5 (15.63%)	2 (5.41%)	3 (11.11%)
Vomiting or nausea^a^ (*n* = 422)	38 (11.69%)	8 (25.00%)	5 (13.51%)	4 (14.29%)
Diarrhoea^a^ (*n* = 416)	38 (11.84%)	8 (25.00%)	5 (13.89%)	6 (22.22%)
Fever (≥ 38°C)^a^ (*n* = 415)	149 (46.86%)	17 (53.13%)	13 (35.14%)	14 (50.00%)
Chills* (*n* = 415)	142 (44.51%)	21 (65.63%)	18 (50.00%)	13 (46.43%)
Headache^a^(*n* = 422)	176 (53.99%)	28 (87.50%)	19 (51.35%)	18 (66.67%)
Joint pain^a^ (*n* = 416)	129 (40.31%)	24 (75.00%)	20 (54.05%)	16 (59.26%)
Myalgia^a^ (*n* = 415)	158 (48.77%)	25 (80.65%)	23 (62.16%)	21 (75.00%)
Change or loss of smell^a^ (*n* = 419)	62 (19.14%)	11 (35.48%)	13 (36.11%)	11 (39.29%)
Change or loss of taste^a^ (*n* = 421)	59 (18.10%)	10 (32.26%)	10 (27.78%)	11 (39.29%)
Fatigue or weakness^a^ (*n* = 419)	176 (54.66%)	28 (87.50%)	25 (67.57%)	20 (71.43%)
Any symptom^a^	303 (92.66%)	32 (100.00%)	35 (94.59%)	27 (96.43%)

^a^the category represented is “yes”

## Discussion

In this study, we aimed to identify symptom clusters 9 and 12 months after a SARS-CoV-2 positive test and describe these clusters regarding symptoms, background demographic and clinical characteristics. Four clusters were identified at 9 and 12 months – no or minor symptoms, joint pain, multi-symptoms, and neurocognitive-related symptoms. Symptom’s frequencies and individuals’ characteristics fluctuated between the two time points.

Our findings complement the literature analysing clusters of Long COVID symptoms [9–15, 24–28]. The cluster with no or minor symptoms (cluster 1) was the largest cluster in our study, comprising 60% of our study population, which is in line with similar studies where this cluster was also the largest, although with no distinctive characteristics [8, 14]. In 2023, Kenny et al. also identified this cluster, however, the most frequent symptoms between Long COVID phenotypes differed depending on the SARS-CoV-2 variant at the time of infection: in the Alpha period, anosmia was more frequent in this cluster, whereas in the Omicron period, no single symptom predominated [29]. During our study period, Omicron was the predominant variant [30], and fatigue was the only symptom that stood out as more frequent but with minor differences from the other symptoms, which is in line with the aforementioned findings. Gerritzen et al. [15] and van den Houdt et al. [13] also observed a cluster with no or minor symptoms, although it included fewer individuals compared to the analogous cluster in our study.

The cluster characterised by joint pain included fewer individuals, which was also found in earlier studies [8, 10, 28]. Individuals in this cluster also had a higher frequency of joint pain and myalgia at the time of the test compared to the other clusters. Tobacco consumption was higher in this cluster, aligning with evidence suggesting a link between this habit and Long COVID pain-related symptoms [31]. Asthma was the pre-existing health condition most frequent in this cluster at 9 months, and at 12 months, asthma was more frequent in multi-symptoms cluster. This disease was associated with general fatigue in Long COVID by a cohort study in Japan [32] and, in fact, fatigue was among the most frequent symptoms reported in both clusters at both times, which seems to confirm this association. A clustering study found higher rates of diabetes and hypertension in this cluster [26], which was not the case in our study. The different magnitude of the studies (national survey vs. regional survey) or the period analysed (from < 1 month until > 6 months after SARS-CoV-2 positive test vs. 9 months) could be possible sources for this difference. Goldhaber et al. have also shown that older individuals were more likely to be in this cluster, however in our study, this was not the cluster with the highest median age at 9 months [11]. That study included individuals who tested positive for SARS-CoV-2 within 1 year period and of which one third had been hospitalised. Our study was community based, included individuals who tested positive for SARS-CoV-2 within 1 month period and less than 1% were hospitalised and individuals hospitalized due to SARS-CoV-2 infection tend to have higher rates of chronic diseases, namely diabetes [33]. Infection severity seems to be a shaping factor for Long COVID symptoms clustering by influencing the rates and specificity of Long COVID symptoms.

The multi-symptoms cluster was characterised by higher frequency of symptoms across several organs with no distinct organ affected, which was also identified in other studies [9, 12, 14, 24, 25, 34]. All the individuals in this cluster reported experiencing fatigue at the time of infection and at 9 and 12 months, which is in line with a previous study where fatigue was also the most common symptom in this cluster [9, 14, 25]. Individuals in this cluster also experienced a higher frequency of symptoms during the SARS-CoV-2 infection and had more pre-existing health conditions. This is in line with van den Houdt et al., which demonstrated that reporting a greater number of acute COVID-19 symptoms was associated with higher odds of belonging to the cluster with a higher prevalence of symptoms [13], and with Nayani et al. which demonstrated that individuals with a history of chronic diseases were more likely to be in this cluster [9]. Additionally, more females and older individuals were in this cluster, which is also shown in other studies [9, 14, 24, 34]. This might be related to other cluster characteristics, namely higher frequency of pre-existing health conditions since older individuals might be more likely to have other health conditions, however this association was not explored in this study. In our study, this cluster had the highest frequency of individuals with a previous infection of SARS-CoV-2, which is in line with previous findings that reinfection is associated with increased prevalence of Long COVID symptoms [26].

Neurocognitive-related symptoms cluster was the second largest cluster in our study. This cluster had been also identified in a meta-analysis with a pooled prevalence of 72% [7]. There are differences in the most frequent symptoms and even in the symptoms that comprise this cluster. In our study, at 9 months, the most frequent symptoms were memory loss and concentration issues, which were also identified in this cluster in other studies [9, 11, 14, 27]. However, studies also report higher frequencies of headache, insomnia, tingling or anosmia [7, 24]. A cardiorespiratory/cardiovascular cluster was also identified in the meta-analysis [7], comprising fatigue, dyspnoea, chest pain, myalgia, headache, and palpitations. In our study, fatigue was the most prevalent symptom and was distributed across clusters, namely no or minor symptoms, joint pain, and multi-symptoms; myalgia were more prevalent in joint pain cluster; and chest pain, headache, palpitations were more prevalent in multi-symptoms cluster. In this study, dyspnoea was also more frequent in multi-symptoms. The most prevalent pre-existing health condition in this cluster seems to be hypertension [24], which was the most prevalent pre-existing health condition in the multi-symptoms cluster found in this study, suggesting that this two may be related or overlapping. Additionally, individuals in this cluster appear to have experienced severe cases of SARS-CoV-2 infection, whereas in our study individuals had mainly mild cases [8].

A systematic review that analysed symptom clusters show that most symptoms tend to decrease in prevalence over time [7], which is in line with our study. Although evidence is not always consistent, since studies show that some symptoms can remain or increase over time or even appear later on [7, 35–37]. The same systematic review also showed that post-exertional malaise continued to increase up to 1 year after SAR-CoV-2 infection [7]. Post-exertional malaise has been commonly reported by individuals with Long COVID and is characterised by fatigue- and pain-related symptoms following even minor physical or mental exercise [38]. In our study, fatigue remained relevant in multi-symptom clusters and even increased its frequency in the joint pain cluster between 9 and 12 months. Also, a prospective study showed that “feeling slowed down” and fatigue were less likely to improve at 12 months in older individuals [36], which was observed in our study. In fact, fatigue was the more frequent symptom at 12 months and, in Long COVID, it can encompass a variety of complaints, such as “brain fog” that can include concentration issues and memory impairments [39]. Memory loss and concentration issues increased their prevalence within the neuro-cognitive cluster. A cohort study also showed that the proportion of memory and cognitive impairment continues to increase up to 24 months after infection [37], which highlights the continued and future relevance of Long COVID as a public health challenge and the need for further reference. Memory loss and concentration issues are often associated with older age, as these faculties can begin to decline over time. Nevertheless, emerging evidence suggests that memory loss can manifest as a post-COVID symptom, affecting even younger demographics. Matias-Guiu et al. [40] found that cognitive issues were more prevalent among younger individuals with lower levels of education and Herrera et al. [41] demonstrated that, among predominantly mild cases of acute SARS-CoV-2 infection, cognitive issues appeared to be more frequent and severe in younger patients. Our sample primarily consists of middle-aged and older individuals, which could bias the findings of this cluster. However, studies that have also identified this cluster report a similar average age [9, 11, 14, 27].

Continuing to explore Long COVID symptoms, delving into specific symptoms and their evolution could assist public health authorities in directing prevention campaigns towards those at higher risk. Additionally, studying specific clusters may help developing evidence-based interventions and therapies as well as enable healthcare providers to intervene early, potentially mitigating the severity of long COVID symptoms and improving patient outcomes.

It is important to note that our study has certain limitations that must be considered. In our study, we defined “Long COVID symptoms” as having ≥ 1 symptom 9 or 12 months after the SARS-CoV-2 test. Given that Long COVID is a diagnosis of exclusion, we and most studies do not exclude other potential conditions. Different methodological approaches, namely regarding symptom assessment and statistical analysis, were found, which might lead to different estimates. Also, although participants were asked about symptoms they experienced in the last 7 days and that they had not experienced before the SARS-CoV-2 test, reported symptoms could be due to other conditions. Symptoms were self-reported by participants and relied on their understanding of the questions being asked, memory of events, symptom definition and valorisation. We tried to use plain language and omit medical terms. Perhaps, with increasing identification of various complications over time, symptoms such as “balance issues” and “palpitations” can be linked to autonomic dysfunction and postural orthostatic tachycardia syndrome (POTS) that might be triggered by COVID-19 infection or COVID-19 vaccination [42], while “memory loss” or “concentration difficulties” have come to be recognised as post-COVID ‘brain fog’. Also, despite adding relevant knowledge to Long COVID condition our study did not cover severe cases of SARS-CoV-2 infection. The severity of the infection appears to influence the frequency and specificity of Long COVID symptoms, thus playing a role in how these symptoms cluster. Another limitation was the lack of information on vaccination due to limited recall at the time of data collection [43, 44]. Linkage COVID-19 vaccination data could be employed in future studies. Although this is not a straightforward approach due to ethical issues, it could also be used as an addition to self-reported symptoms, which are an important indicator of individual’s health behaviours. Although a 3-months follow-up can provide valuable insights into symptom clusters evolution, it may not be sufficient to fully capture the long-term progression and variability of Long COVID symptoms, which could affect the observed patterns. Future research with extended follow-up periods would be beneficial to gain a more comprehensive understanding of the persistence and fluctuation of symptoms over time.

To the best of our knowledge, this is the first study accessing Long COVID symptom clusters at 9 and 12 months, which adds relevant evidence from a European country and to the evolution of symptoms over time. Another important strength of our study is the exploration of clustering of an extensive range of Long COVID self-reported symptoms based on the International Severe Acute Respiratory and Emerging Infection Consortium (ISARIC) / WHO COVID-19 clinical characterisation [45]. This initiative aims to prevent illness and deaths from infectious disease outbreaks by offering a skilled and coordinated research response through a global federation of clinical research networks. This study also covered the Omicron phase and encompassed a broad spectrum of community-based cases, including both PCR and rapid antigen tests. This inclusive methodology contrasts with many studies that focus solely on healthcare-seeking populations or pre-Omicron phases, allowing for a more representative analysis of Long COVID across different detection methods and timeframes.

The findings of the current study add important evidence to the body of work regarding the heterogeneity of Long COVID symptoms. Given the public health relevance that Long COVID has and will continue to have in the foreseeable future, it is important to continue deepening the research on this issue. Future studies should include larger samples to analyse the risk factors for the different clusters. Longer periods of infection should also be analysed to cover different variants and explore how they influence Long COVID symptom clusters.

## Conclusion

We identified four clusters of symptoms within individuals who reported having one or more symptoms at 9 and 12 months after a positive SARS-Cov-2 infection. Clusters remained similar at 9 and 12 months, except for a slight variation in the frequency of symptoms that composed each cluster. All symptoms decreased frequency over time, however, within the neuro-cognitive cluster, memory loss and concentration issues increased their frequency at 12 months. Analysing Long COVID symptoms cluster could help to identify treatments for this condition which will remain a relevant public health issue in the years to come. Hence, further validation of the observed clusters and analysis of its risk factors is needed.

## Supplementary Information

Supplementary Material 1.

Supplementary Material 2.

Supplementary Material 3.

## Data Availability

Data are available upon reasonable request.
