# Correction: Long COVID is not the same for everyone: a hierarchical cluster analysis of long COVID symptoms 9 and 12 months after SARS-CoV-2 test

**DOI:** 10.1186/s12879-024-10086-9

**Published:** 2024-10-18

**Authors:** Marta Moniz, Carolina Ruivinho, Ana Rita Goes, Patrícia Soares, Andreia Leite

**Affiliations:** 1 Public Health Research Centre, Comprehensive Health Research Center, NOVA National School of Public Health, CHRC, REAL, CCAL, NOVA University Lisbon, Lisbon, Portugal; 2 National Institute of Health Doutor Ricardo Jorge, Lisbon, Portugal

**Correction: BMC Infect Dis. 2024 Sep 19;24(1):1001**

10.1186/s12879-024-09896-8

Following publication of the original article [1], we were notified that a few instances of “RT-PCR SARS-CoV-2 test” should actually read “SARS-CoV-2 test”.

**Methods (Abstract)**:

Original text: “RT-PCR SARS-CoV-2 test”.

Correction: This should read “SARS-CoV-2 test”.

**Methods (Main Text)**:

Original text: “Individuals who had a positive RT-PCR SARS-CoV-2 test in August 2022, (…)”.

Correction: This should read “Individuals who had a positive SARS-CoV-2 test in August 2022 (both PCR and rapid antigen test), (…)”.

Current text: “1) names and contact numbers (landline or mobile) of a random sample of 10,000 individuals who had a RT-PCR SARS-CoV-2 test, (…)”.

Correction: This should read “1) names and contact numbers (landline or mobile) of a random sample of 10,000 individuals who had a SARS-CoV-2 test, (…)”.

The original article has been corrected.
