# Supplementary material for: Long COVID is not the same for everyone: a hierarchical cluster analysis of Long COVID symptoms 9 and 12 months after SARS-CoV-2 test
Source: BMC Infect Dis. 2024 Sep 19;24:1001. doi: 10.1186/s12879-024-09896-8 (PMC11412022; doi:10.1186/s12879-024-09896-8)
Supplement: Supplementary file 1 — Supplementary Material 1. [file 12879_2024_9896_MOESM1_ESM.pdf]

Table 1: Characterization of participants' sociodemographic and clinical characteristics at 9 and 12 months after the SARS-CoV-2 positive test.

	9 months follow-up	12 months follow-up
**Variable**	**N = 552**	**N = 424**
**Age**		
Mean (Range)	52.62 (18.00, 92.00)	53.18 (18.00, 92.00)
Median (IQR)	52.00 (40.75, 64.00)	53.00 (41.75, 66.00)
**Sex**		
Female	320 (58.61%)	247 (59.09%)
Male	226 (41.39%)	171 (40.91%)
Missing	6	6
**Tobacco consumption**		
No	435 (78.95%)	340 (80.19%)
Yes	116 (21.05%)	84 (19.81%)
Missing	1	---
**Alcohol consumption**		
Never	153 (27.72%)	123 (29.01%)
2 to 4 times a month or less	258 (46.74%)	195 (45.99%)
2 times a week or more	141 (25.54%)	106 (25.00%)
**Overweight**		
No	243 (44.67%)	186 (44.50%)
Yes	301 (55.33%)	232 (55.50%)
Missing	8	6
**Pre-existing health conditions**		
**COVID-19**		
No	422 (77.72%)	324 (78.07%)
Yes	121 (22.28%)	91 (21.93%)
Missing	9	9
**Hypertension**		
No	387 (70.36%)	290 (68.72%)
Yes	163 (29.64%)	132 (31.28%)
Missing	2	2
**Diabetes**		
No	496 (90.35%)	375 (89.07%)
Yes	53 (9.65%)	46 (10.93%)
Missing	3	3
**High cholesterol**		
No	391 (71.22%)	288 (68.25%)
Yes	158 (28.78%)	134 (31.75%)

	9 months follow-up	12 months follow-up
**Variable**	**N = 552**	**N = 424**
Missing	3	2
**Asthma**		
No	501 (91.09%)	381 (90.28%)
Yes	49 (8.91%)	41 (9.72%)
Missing	2	2
**Bronchitis**		
No	527 (95.82%)	405 (95.97%)
Yes	23 (4.18%)	17 (4.03%)
Missing	2	2
**Pulmonary fibrosis**		
No	547 (99.64%)	419 (99.52%)
Yes	2 (0.36%)	2 (0.48%)
Missing	3	3
**Heart failure**		
No	523 (95.09%)	403 (95.50%)
Yes	27 (4.91%)	19 (4.50%)
Missing	2	2
**Reflux disease**		
No	489 (88.91%)	377 (89.34%)
Yes	61 (11.09%)	45 (10.66%)
Missing	2	2
**Psychiatric condition**		
No	456 (83.06%)	341 (81.00%)
Yes	93 (16.94%)	80 (19.00%)
Missing	3	3
**Myocardial infarction**		
No	534 (97.45%)	411 (97.62%)
Yes	14 (2.55%)	10 (2.38%)
Missing	4	3
**Stroke**		
No	537 (97.99%)	411 (97.62%)
Yes	11 (2.01%)	10 (2.38%)
Missing	4	3
**Thrombosis**		
No	540 (98.54%)	416 (99.05%)
Yes	8 (1.46%)	4 (0.95%)
Missing	4	4

	9 months follow-up	12 months follow-up
**Variable**	**N = 552**	**N = 424**
**Pulmonary thrombosis**		
No	544 (99.45%)	418 (99.76%)
Yes	3 (0.55%)	1 (0.24%)
Missing	5	5
**Any condition**		
No	150 (27.17%)	105 (24.76%)
Yes	402 (72.83%)	319 (75.24%)
**Symptoms experienced in the 7 days before the SARS-CoV-2 test**		
**Cough**		
No	305 (55.86%)	228 (54.42%)
Yes	241 (44.14%)	191 (45.58%)
Missing	6	5
**Breathing difficulties**		
No	448 (81.60%)	342 (80.85%)
Yes	101 (18.40%)	81 (19.15%)
Missing	3	1
**Runny nose**		
No	255 (47.13%)	186 (44.93%)
Yes	286 (52.87%)	228 (55.07%)
Missing	11	10
**Sore throat**		
No	263 (48.35%)	197 (47.02%)
Yes	281 (51.65%)	222 (52.98%)
Missing	8	5
**Chest pain**		
No	489 (90.06%)	383 (91.41%)
Yes	54 (9.94%)	36 (8.59%)
Missing	9	5
**Abdominal pain**		
No	505 (92.49%)	386 (91.69%)
Yes	41 (7.51%)	35 (8.31%)
Missing	6	3
**Vomiting or nausea**		
No	480 (87.59%)	367 (86.97%)
Yes	68 (12.41%)	55 (13.03%)
Missing	4	2
**Diarrhoea**		

	9 months follow-up	12 months follow-up
**Variable**	**N = 552**	**N = 424**
No	477 (87.85%)	359 (86.30%)
Yes	66 (12.15%)	57 (13.70%)
Missing	9	8
**Fever (≥38°C)**		
No	293 (54.36%)	222 (53.49%)
Yes	246 (45.64%)	193 (46.51%)
Missing	13	9
**Chills**		
No	291 (53.89%)	221 (53.25%)
Yes	249 (46.11%)	194 (46.75%)
Missing	12	9
**Headache**		
No	230 (41.89%)	181 (42.89%)
Yes	319 (58.11%)	241 (57.11%)
Missing	3	2
**Joint pain**		
No	294 (54.24%)	227 (54.57%)
Yes	248 (45.76%)	189 (45.43%)
Missing	10	8
**Myalgia**		
No	245 (44.79%)	193 (45.95%)
Yes	302 (55.21%)	227 (54.05%)
Missing	5	4
**Change or loss of smell**		
No	413 (75.78%)	322 (76.85%)
Yes	132 (24.22%)	97 (23.15%)
Missing	7	5
**Change or loss of taste**		
No	423 (77.33%)	331 (78.62%)
Yes	124 (22.67%)	90 (21.38%)
Missing	5	3
**Fatigue or weakness**		
No	220 (40.15%)	170 (40.57%)
Yes	328 (59.85%)	249 (59.43%)
Missing	4	5
**Any symptom**		
No	37 (6.70%)	27 (6.37%)

	9 months follow-up	12 months follow-up
**Variable**	**N = 552**	**N = 424**
Yes	515 (93.30%)	397 (93.63%)
**Symptoms experienced months after positive SARS-CoV-2 test**		
**Persistent or worsening of usual cough**		
No	509 (92.21%)	392 (92.45%)
Yes	43 (7.79%)	32 (7.55%)
**Breathing difficulties**		
No	519 (94.02%)	403 (95.05%)
Yes	33 (5.98%)	21 (4.95%)
**Runny nose**		
No	489 (88.59%)	389 (91.75%)
Yes	63 (11.41%)	35 (8.25%)
**Sore throat**		
No	527 (95.47%)	400 (94.34%)
Yes	25 (4.53%)	24 (5.66%)
**Chest pain**		
No	524 (94.93%)	405 (95.52%)
Yes	28 (5.07%)	19 (4.48%)
**Abdominal pain**		
No	533 (96.56%)	416 (98.11%)
Yes	19 (3.44%)	8 (1.89%)
**Vomiting or nausea**		
No	536 (97.10%)	415 (97.88%)
Yes	16 (2.90%)	9 (2.12%)
**Diarrhoea**		
No	525 (95.11%)	417 (98.35%)
Yes	27 (4.89%)	7 (1.65%)
**Fever (≥38)**		
No	550 (99.64%)	421 (99.29%)
Yes	2 (0.36%)	3 (0.71%)
**Chills**		
No	547 (99.09%)	417 (98.35%)
Yes	5 (0.91%)	7 (1.65%)
**Headache**		
0	487 (88.22%)	392 (92.45%)
1	65 (11.78%)	32 (7.55%)
**Joint pain**		
No	454 (82.25%)	380 (89.62%)

	9 months follow-up	12 months follow-up
**Variable**	**N = 552**	**N = 424**
Yes	98 (17.75%)	44 (10.38%)
**Myalgia**		
No	496 (89.86%)	383 (90.33%)
Yes	56 (10.14%)	41 (9.67%)
**Change or loss of smell**		
No	527 (95.47%)	411 (96.93%)
Yes	25 (4.53%)	13 (3.07%)
**Change or loss of taste**		
No	533 (96.56%)	411 (96.93%)
Yes	19 (3.44%)	13 (3.07%)
**Fatigue or weakness**		
No	436 (78.99%)	353 (83.25%)
Yes	116 (21.01%)	71 (16.75%)
**Pain breathing**		
No	542 (98.19%)	415 (97.88%)
Yes	10 (1.81%)	9 (2.12%)
**Palpitations**		
No	511 (92.57%)	406 (95.75%)
Yes	41 (7.43%)	18 (4.25%)
**Loss of appetite**		
No	533 (96.56%)	418 (98.58%)
Yes	19 (3.44%)	6 (1.42%)
**Constipation**		
No	521 (94.38%)	412 (97.17%)
Yes	31 (5.62%)	12 (2.83%)
**Difficulties urinating**		
No	544 (98.55%)	422 (99.53%)
Yes	8 (1.45%)	2 (0.47%)
**Swollen ankle**		
No	512 (92.75%)	403 (95.05%)
Yes	40 (7.25%)	21 (4.95%)
**Balance issues**		
No	510 (92.39%)	400 (94.34%)
Yes	42 (7.61%)	24 (5.66%)
**Not feeling one side of the body or face**		
No	549 (99.46%)	421 (99.29%)
Yes	3 (0.54%)	3 (0.71%)

	9 months follow-up	12 months follow-up
**Variable**	**N = 552**	**N = 424**
**Tingling**		
No	494 (89.49%)	398 (93.87%)
Yes	58 (10.51%)	26 (6.13%)
**Fainting**		
No	549 (99.46%)	421 (99.29%)
Yes	3 (0.54%)	3 (0.71%)
**Seizures**		
No	552 (100.00%)	424 (100.00%)
**Tremors**		
No	548 (99.28%)	423 (99.76%)
Yes	4 (0.72%)	1 (0.24%)
**Chewing difficulties**		
No	546 (98.91%)	419 (98.82%)
Yes	6 (1.09%)	5 (1.18%)
**Swallowing difficulties**		
No	551 (99.82%)	424 (100.00%)
Yes	1 (0.18%)	---
**Tinnitus**		
No	514 (93.12%)	406 (95.75%)
Yes	38 (6.88%)	18 (4.25%)
**Insomnia**		
No	479 (86.78%)	387 (91.27%)
Yes	73 (13.22%)	37 (8.73%)
**Rash**		
No	539 (97.64%)	417 (98.35%)
Yes	13 (2.36%)	7 (1.65%)
**Concentration issues**		
No	468 (84.78%)	384 (90.57%)
Yes	84 (15.22%)	40 (9.43%)
**Memory loss**		
No	417 (75.54%)	356 (83.96%)
Yes	135 (24.46%)	68 (16.04%)
