# Supplementary material for: Long COVID is not the same for everyone: a hierarchical cluster analysis of Long COVID symptoms 9 and 12 months after SARS-CoV-2 test
Source: BMC Infect Dis. 2024 Sep 19;24:1001. doi: 10.1186/s12879-024-09896-8 (PMC11412022; doi:10.1186/s12879-024-09896-8)
Supplement: Supplementary file 2 — Supplementary Material 2. [file 12879_2024_9896_MOESM2_ESM.pdf]

First questionnaire

1. General information about the participant

**Are you male or female?**<br>- Male<br>- Female<br>- Another _________<br>- You do not want to answer
**What is the highest level of education you have completed?**<br>- Can neither read nor write<br>- Does not have the 1st cycle of basic education (4th year)<br>- 1st cycle of basic education (4th year)<br>- 2nd cycle of basic education (6th year)<br>- 3rd cycle of basic education (9th year)<br>- Secondary education (12th grade) or post-secondary non-tertiary education;<br>- Higher education (Short term, Bachelor, Degree, Master or Doctorate)<br>- You do not want to answer
**What is your current professional situation? I will read you a list of options and I would like you to answer with the option that best describes your situation:**<br>- Works<br>- You are unemployed<br>- He is a student<br>- You are a working student<br>- You are retired<br>- Is engaged in unpaid domestic work (is a domestic worker or informal caregiver)<br>- You are permanently incapacitated (unable to work)<br>- Another<br>- You do not want to answer
***If the participant answered that he/she "Works", "Is a student" or "Is a working-student": Was there a change in the duration, in hours of work or school, compared to the situation he/she was in 9 months ago, in August 2022?**<br>- Yes<br>- No<br>- You do not want to answer
***If the answer to the previous question is <u>YES</u>: Was there an increase, decrease or did you need to stop?**

- Increase<br>- Decrease<br>- Need to stop<br>- Unknown<br>- You do not want to answer
***If the answer to the previous question is DIMINING or NEEDING TO STOP: What is the reason for the decrease or need to stop? I will read you a list of options and I would like you to respond with the option that best describes your situation or explain the reason:**<br>- Poor health;<br>- New carer status;<br>- Restrictions due to pandemic COVID-19;<br>- Outra ________________________

"Still bearing in mind August 2022 at the time of that test, I would now like to ask you some questions about your habits and your health at that time."

**Did you smoke?**<br>- Yes<br>- No<br>- You do not want to answer
***If the answer to the question "Did you smoke?" is YES: How many cigarettes did you smoke per day, on average?**<br>____
***If the answer to the question "Did you smoke?" is YES: Approximately how many years ago had you started smoking?**<br>____
**How often did you consume alcoholic drinks? I will read you a list of options and I would like you to respond with the one that you think best describes your situation:**<br>- Never<br>- Once a month or less<br>- 2 to 4 times a month<br>- 2 to 3 times a week<br>- 4 or more times a week<br>- You do not want to answer

***If the answer to the previous question is <u>NO or DO NOT WANT TO ANSWER</u>: On the days you were drinking, how many drinks did you consume on average? I will read you a list of options and I would like you to respond with the one that you think best describes your situation:**<br>- 1 or 2<br>- 3 or 4<br>- 5 or 6<br>- 7 a 9<br>- 10 or more drinks<br>- You do not want to answer
**Did you practice more than 30 minutes daily of physical activity 3 or more times a week?**<br>- Yes<br>- No<br>- You do not want to answer
**#Now I will read you a list of diseases or health disorders and I would like you to tell me if before August 2022 you have been diagnosed with any of them:**<br>1. **COVID-19** Yes/No/Don't know/wouldn't answer<br>1.1. **If <u>yes</u>: On what date were you diagnosed? ____________**<br>2. **Hypertension** Yes/No/does not want to answer<br>3. **Diabetes (other than diabetes during pregnancy)** Yes/No/ Don't know/ Don't want to answer<br>4. **High Cholesterol** Yes/No/ Don't know/ Don't want to **answer**<br>5. **Asthma** Yes/No/don't know/no answer<br>6. **Chronic bronchitis or pulmonary emphysema** Yes/No/ Don't know/ Don't want to answer<br>7. **Pulmonary fibrosis** Yes/No/don't know/no answer<br>8. **Heart failure** Yes/No/don't know/no answer<br>*9.* **Reflux disease** Yes/No/don't know/no answer<br>10. **Any psychological illness (e.g. anxiety disorder or depression)** Yes/No/Does not know/does not want to answer<br>11. **Heart attack** Yes/No/Don't know/No answer<br>12. **Heart attack** Yes/No/Does not know/does not want to answer<br>13. **Deep vein thrombosis (e.g. thrombosis on one leg)** Yes/No/ Don't know/ Don't want to answer<br>14. **Pulmonary thromboembolism (thrombosis in the lung)** Yes/No/ Don't know/ Don't want to answer

**How tall are you?**<br><br>____
**What is your weight at today's date? If you do not know the exact figure, please answer an approximate value.**<br><br>____
**At the time of the test, in August 2022, was your weight the same as you answered in the previous question?**<br>- Yes<br>- No<br>- Don't know<br>- You do not want to answer
***If the answer to the previous question is NO: What was your weight at the time of the test in August 2022? If you do not know the exact value, please answer an approximate value.**<br><br>____

"Continuing still in August 2022, at the time of when you took that test, I would like you to answer some questions about your symptoms."

2. Symptoms experienced at the time of testing

**I will read you a list of symptoms and I would like you to tell me whether or not you experienced any or some of them at the time of the test in August 2022. As you hear each symptom I would ask you to answer only yes, no or do not remember:**<br>1. **Persistent cough or worsening of your usual cough** Yes/No/ Don't know/ Don't want to answer<br>2. **Difficulty in breathing** Yes/No/Don't know/No answer<br>3. **Runny or runny nose** Yes/No/ Don't know/ Don't want to answer<br>4. **Sore throat** Yes/No/don't know/no answer<br>5. **Chest pain** Yes/No/ Don't know/ Don't want to answer<br>6. **Belly ache** Yes/No/ Don't know/ Don't want to answer<br>7. **Vomiting or nausea** Yes/No/ Don't know/ Don't want to answer<br>8. **Diarrhoea** Yes/No/ Don't know/ Don't want to answer<br>9. **Fever over 38°C on thermometer** Yes/No/ Don't know/ Don't want to answer<br>10. **Chills** Yes/No/don't know/no answer<br>11. **Headache** Yes/No/don't know/no answer

12. **Joint pain** Yes/No/don't know/no answer
13. **Muscle pain in the whole body** Yes/No/Don't know/doesn't want to answer
14. **Change in smell** Yes/No/don't know/no answer
15. **Change in taste** Yes/No/ Don't know/ Don't want to answer
16. **Tiredness or lack of strength** Yes/No/don't know/no answer

***If you answered YES to the symptom Tiredness or lack of strength: On an intensity from 0 to 10, with 0 being no tiredness and 10 being extreme tiredness, how would you rate the intensity of your tiredness 9 months ago at the time of the test in August 2022:**

____

I will read you 4 options and I would like you to tell me which statement best described your feeling of breathlessness 9 months ago at the time of the test, in August 2022:

- I only experienced shortness of breath during intense physical exercise
- He would get short of breath when rushing or walking down a slightly sloping road
- He walked slower than other people because of shortness of breath, or had to stop for breath when walking at his normal pace
- I would stop for breath after walking 100 metres or after a few minutes
- felt too tired or out of breath to leave the house, get dressed or undressed
- You do not want to answer

***Question only if the participant is from the POSITIVE TEST group: During the period of illness, I would like to know what level of health care you needed. For this, I am going to give you 5 options and I would like you, at the end, to choose the most suitable option by thinking of the moment when you felt worst during the whole episode:**

- Did not seek any care
- You went to the health centre or clinic for an appointment or actively sought an appointment by telephone
- He went to the emergency service
- You have been hospitalised
- Was admitted to intensive care
- You do not want to answer

We are trying to find out how you feel about your health. I will explain what to do as I go along, but stop me if you don't understand something or if things are not clear to you. There are no right or wrong answers. We are only interested in your personal view.

First, I will read out some questions. Each question has five choices. Tell me which answer best describes your health in the month before you took the PCR test, in July 2022.

First I'd like to ask you about mobility. Would you say that:

- You have no problems in walking about?
- You have slight problems in walking about?
- You have moderate problems in walking about?
- You have severe problems in walking about?
- You are unable to walk about?
- You do not want to answer

Next I'd like to ask you about self-care. Would you say that:

- You have no problems washing or dressing yourself?
- You have slight problems washing or dressing yourself?
- You have moderate problems washing or dressing yourself?
- You have severe problems washing or dressing yourself?
- You are unable to wash or dress yourself?
- You do not want to answer

Next I'd like to ask you about usual activities, for example work, study, housework, family or leisure activities. Would you say that:

- You have no problems doing your usual activities?
- You have slight problems doing your usual activities?
- You have moderate problems doing your usual activities?
- You have severe problems doing your usual activities?
- You are unable to do your usual activities?
- You do not want to answer

Next I'd like to ask you about pain or discomfort. Would you say that:

- You have no pain or discomfort?
- You have slight pain or discomfort?
- You have moderate pain or discomfort?
- You have severe pain or discomfort?
- You have extreme pain or discomfort?

- You do not want to answer

Finally I'd like to ask you about anxiety or depression. Would you say that:

- You are not anxious or depressed?
- You are slightly anxious or depressed?
- You are moderately anxious or depressed?
- You are severely anxious or depressed?
- You are extremely anxious or depressed?
- You do not want to answer

Now, I would like to ask you to say how good or bad your health was in the month before you took the PCR test, in July 2022. I'd like you to try to imagine a vertical line numbered from 0 to 100. 100 at the top of the line means the best health you can imagine. 0 at the bottom of the line means the worst health you can imagine. I would now like you to tell me the point on this line where you would put your health in the month before you took the PCR test, in July 2022.

____

"Let's move now to the present moment. In this part of the survey, I would like you to answer some questions about your symptoms and your health today."

3. Symptoms and health of the participant today

***Question only if the participant is from the POSITIVE TEST group: For this question I will read you 5 alternatives and at the end I would like you to tell me which option you most identify with. Do you feel fully recovered from COVID-19?**

- Totally disagree
- I disagree
- I neither agree nor disagree
- I agree
- Totally agree
- You do not want to answer

Now I am going to read you a list of various symptoms and I would like you to tell me whether or not, in the last 7 days, you have felt any or some of them, which you had not felt before the test was taken 9 months ago in August 2022. Some symptoms will be the same and some will be different from those I have already

asked about. As you hear each symptom I would ask you to answer only yes, no or do not remember:

1. **Persistent cough or worsening of usual cough** Yes/No/Don't know/would not answer
2. **Difficulty in breathing** Yes/No/Don't know/would not answer
3. **Runny or runny nose** Yes/No/Don't know/wouldn't answer
4. **Sore throat** Yes/No/Don't know/no answer
5. **Chest pain** Yes/No/Don't know/would not answer
6. **Belly ache** Yes/No/Don't know/wouldn't answer
7. **Vomiting or nausea** Yes/No/Don't know/would not answer
8. **Diarrhoea** Yes/No/Don't know/no answer
9. **Fever with temperature above 38°C on thermometer** Yes/No/Don't know/does not want to answer
10. **Chills** Yes/No/Don't know/No answer
11. **Headache** Yes/No/Don't know/no answer
12. **Joint pain** Yes/No/Don't know/no answer
13. **Muscle aches in the whole body** Yes/No/Don't know/wouldn't answer
14. **Change in smell** Yes/No/Don't know/no answer
15. **Taste change** Yes/No/Don't know/no answer
16. **Tiredness or lack of strength** Yes/No/Don't know/no answer
17. **Pain when breathing** Yes/No/Don't know/No answer
18. **Palpitations (or feeling your heart racing)** Yes/No/Don't know/wouldn't answer
19. **Loss of appetite** Yes/No/Don't know/no answer
20. **Difficulty in evacuating** Yes/No/Don't know/would not answer
21. **Difficulty in passing urine** Yes/No/Don't know/no answer
22. ***If MALE: Erectile dysfunction or unusual difficulties in achieving or maintaining an erection** Yes/No/Don't know/doesn't want to answer
23. ***If female and age <55 years: Changes in your usual menstrual cycle** Yes/No/Don't know/doesn't want to answer
24. **Swollen ankle(s)** Yes/No/Does not know/does not want to answer
25. **Balance problems** Yes/No/don't know/no answer
26. **Inability to feel one side of the body or face** Yes/No/Does not know/does not want to answer
27. **Tingling sensation** Yes/No/Don't know/would not answer
28. **Fainting episode** Yes/No/Don't know/would not answer
29. **Convulsions** Yes/No/Don't know/would not answer
30. **Tremors** Yes/No/Don't know/would not answer
31. **Difficulty in swallowing** Yes/No/don't know/no **answer**

32. **Difficulty in chewing** Yes/No/don't know/no answer
33. **Hearing a buzzing sound for most of a day** Yes/No/Don't know/doesn't want to answer
34. **Insomnia** Yes/No/Don't know/wouldn't answer
35. **Redness of the skin** Yes/No/Don't know/no answer
36. **Difficulty in concentrating** Yes/No/Does not know/does not want to answer
37. **Difficulty in remembering** Yes/No/Does not know/does not want to answer

***If you answered YES to the symptom Tiredness or lack of strength: On an intensity from 0 to 10, with 0 being no tiredness and 10 being extreme tiredness, how would you rate the intensity of your tiredness in the last 24 hours?**

____

***If you answered YES to at least one of the previous symptoms: Have you sought care for any of the symptoms you mentioned?**

- Yes
- No
- You do not want to answer

***If you answered YES to the previous question about seeking care: Have you been diagnosed with the post-COVID-19 condition?**

- Yes
- No
- You do not want to answer

***If you answered YES to at least one of the previous symptoms and answered that you WORK or ARE WORK-STUDENT in the question on work status: During the past seven days, to what extent have the symptoms you have experienced affected your productivity while at work? Please select a number from 0 to 10, where 0 is no effect on your work and 10 has completely prevented you from doing your work.**

____

***If you answered YES to at least one of the previous symptoms and answered that you WORK or ARE WORK-STUDENT in the question on work status: During the past seven days, to what extent have the symptoms you have experienced affected your ability to carry out your normal non-work related daily activities? Please select a number from 0 to 10, where 0 corresponds to no effect on daily activities and 10 has completely prevented you from carrying out your daily activities.**

____

I am going to read you 4 options and I would like you to tell me which statement best describes your feeling of breathlessness TODAY:

- I only experienced shortness of breath during intense physical exercise
- He would get short of breath when rushing or walking down a slightly sloping road
- He walked slower than other people because of shortness of breath, or had to stop for breath when walking at his normal pace
- I would stop for breath after walking 100 metres or after a few minutes
- felt too tired or out of breath to leave the house, get dressed or undressed
- You do not want to answer

Now I will repeat the questions I asked you earlier about your state of health. We want to find out what you think about your health TODAY.

First I'd like to ask you about mobility. Would you say that:

- You have no problems in walking about?
- You have slight problems in walking about?
- You have moderate problems in walking about?
- You have severe problems in walking about?
- You are unable to walk about?
- You do not want to answer

Next I'd like to ask you about self-care. Would you say that:

- You have no problems washing or dressing yourself?
- You have slight problems washing or dressing yourself?
- You have moderate problems washing or dressing yourself?
- You have severe problems washing or dressing yourself?
- You are unable to wash or dress yourself?
- You do not want to answer

Next I'd like to ask you about usual activities, for example work, study, housework, family or leisure activities. Would you say that:

- You have no problems doing your usual activities?
- You have slight problems doing your usual activities?
- You have moderate problems doing your usual activities?
- You have severe problems doing your usual activities?
- You are unable to do your usual activities?
- You do not want to answer

Next I'd like to ask you about pain or discomfort. Would you say that:

- You have no pain or discomfort?
- You have slight pain or discomfort?
- You have moderate pain or discomfort?
- You have severe pain or discomfort?
- You have extreme pain or discomfort?
- You do not want to answer

Finally I'd like to ask you about anxiety or depression. Would you say that:

- You are not anxious or depressed?
- You are slightly anxious or depressed?
- You are moderately anxious or depressed?
- You are severely anxious or depressed?
- You are extremely anxious or depressed?
- You do not want to answer

Going back to the idea of the vertical line numbered from 0 to 100, with 100 being the best health you can imagine and 0 being the worst health you can imagine, I would ask you to tell me where on this scale you would place your health TODAY.

____

From the date of the test until today, have you been diagnosed with any of these diseases? I will read out a list of diseases and would like you to answer Yes or No:

1. **COVID-19** Yes/No/Don't know/wouldn't answer
   1.1. **If <u>YES</u>: On what date were you diagnosed?** _____________
2. ***If you answered NO to question #: High Voltage** Yes/No/Don't know/wouldn't answer
3. ***If you answered NO to question #: Diabetes, excluding diabetes during pregnancy** Yes/No/Don't know/wouldn't answer
4. ***If you answered NO to question #: Asthma** Yes/No/Don't know/wouldn't answer
5. ***If you answered NO to question #: Chronic Bronchitis** Yes/No/Don't know/wouldn't answer
6. **Stroke (or heart attack)** Yes/No/Don't know/wouldn't answer
7. **Stroke** Yes/No/Don't know/would not answer

8. **Deep vein thrombosis (e.g. thrombosis on one leg)** Yes/No/Don't know/would not answer
9. **Pulmonary thromboembolism (thrombosis in the lung)** Yes/No/Don't know/would not answer

Have you been diagnosed with any other illness that has not been mentioned?

- Yes. **Which one?** ________________________
- No
- You do not want to answer

Did you receive the vaccine against COVID-19? Please answer yes or no.

- Yes
- No
- You do not want to answer

***If the answer to the previous question is YES: How many doses of vaccine did you receive?**

- 1
- 2
- 3
- You do not want to answer

***If the answer to the question " How many doses of vaccine did you receive?" is ≥ 1: When did you receive your first dose?**

____

*** If the answer to the question "How many doses of vaccine did you receive?" is ≥ 2: When did you receive the second dose?**

____

*** If the answer to the question "How many doses of vaccine did you receive?" is ≥ 3: When did you receive the third dose?**

____

*** If the answer to the question "How many doses of vaccine did you receive?" is ≥ 4: When did you receive the fourth dose?**

____

*** If the answer to the question "How many doses of vaccine did you receive?" is 5: When did you receive the fifth dose?**

____

*** If the answer to the previous questions was "I do not remembre": When did you receive the <u>last</u> dose?**<br><br>__________________________
**Have you received a flu vaccine in the last 12 months? Please answer yes or no.**<br>- Yes<br>- No<br>- You do not want to answer
**Have you received pneumonia [or pneumococcal] vaccine in the last 5 years? Please answer yes or no.**<br>- Yes<br>- No<br>- You do not want to answer

We will now enter the last phase of the questionnaire. I would like to ask you some questions about your emotional and psychological well-being. There are no right or wrong answers. We are only interested in your personal opinion.

4. Mental Health Scales

(PHQ - Depression Disorder Screening Scale)

**During the last 14 days, on how many of these days have you been affected by any of the following problems?**<br><br>**1. "I had little interest or pleasure in doing things":**<br>→ Never 0<br>→ On several days +1<br>→ On more than half the number of days +2<br>→ On almost every day +3<br>→ You do not want to answer<br><br>**2. "I felt discouraged, discouraged or hopeless"**<br>→ Never 0<br>→ On several days +1<br>→ On more than half the number of days +2<br>→ On almost every day +3

→ You do not want to answer

***If the total score for the last two statements is ≥3, apply the following questions:**

3. **"I had difficulty falling asleep or sleeping uninterruptedly, or I overslept"**

→ Never
→ On several days
→ On more than half the number of days
→ On almost every day
→ You do not want to answer

4. **"I felt tired or lacked energy"**

→ Never
→ On several days
→ On more than half the number of days
→ On almost every day
→ You do not want to answer

5. **"I had a lack or excess of appetite"**

→ Never
→ On several days
→ On more than half the number of days
→ On almost every day
→ You do not want to answer

6. **"I felt that I don't like myself - or that I am a failure or that I have let myself or my family down"**

→ Never
→ On several days
→ On more than half the number of days
→ On almost every day
→ You do not want to answer

7. **"I found it difficult to concentrate on things, like when reading the newspaper or watching TV"**

→ Never
→ On several days

→ On more than half the number of days
→ On almost every day
→ You do not want to answer

8. **"I moved or spoke so slowly that other people might have noticed. Or the opposite: I was agitated to the point of moving around much more than usual.**

→ Never
→ On several days
→ On more than half the number of days
→ On almost every day
→ You do not want to answer

9. **"I thought it would be better to be dead, or to hurt myself in some way"**

→ Never
→ On several days
→ On more than half the number of days
→ On almost every day
→ You do not want to answer

(GAD - Generalised Anxiety Disorder Screening Scale)

During the last 14 days, on how many days have you been affected by any of the following problems?

1. **"I felt nervous, anxious or irritable".**

→ Never 0
→ On several days +1
→ On more than half the number of days +2
→ On almost every day +3
→ You do not want to answer

2. **"I was unable to stop worrying or control the worries"**

→ Never 0
→ On several days +1

→ On more than half the number of days +2
→ On almost every day +3
→ You do not want to answer

***If the total score for the last two statements is ≥3, apply the following questions:**

3. "I worried too much about different issues"
→ Never
→ On several days
→ On more than half the number of days
→ On almost every day
→ You do not want to answer

4. "I found it difficult to relax"
→ Never
→ On several days
→ On more than half the number of days
→ On almost every day
→ You do not want to answer

5. "I was so restless it was hard to be quiet
→ Never
→ On several days
→ On more than half the number of days
→ On almost every day
→ You do not want to answer

6. "I was easily annoyed or irritable"
→ Never
→ On several days
→ On more than half the number of days
→ On almost every day
→ You do not want to answer

7. "I felt fear, as if something terrible might happen"
→ Never
→ On several days

→ On more than half the number of days<br>→ On almost every day<br>→ You do not want to answer

(PC-PTSD - Posttraumatic Stress Disorder Screening Scale)

To conclude the questionnaire, from an emotional and psychological point of view, I want to ask about your experience with the COVID-19 pandemic.

**Sometimes events happen that are strangely or especially frightening, horrible or traumatic.**<br>**Do you consider the COVID-19 pandemic to be such an event?**<br>- Yes<br>- No<br>- You do not want to answer
***If the answer to the previous question is <u>YES</u>, apply the following questions:**<br>**In the past month, have you...**<br>1. **Had nightmares about the pandemic or thought about the pandemic when you did not want to?** Yes/No/does not want to answer<br>2. **Tried hard not to think about the pandemic or went out of your way to avoid situations that reminded them of the pandemic?** Yes/No/does not want to answer<br>3. **Been constantly on guard, watchful, or easily startled?** Yes/No/does not want to answer<br>4. **Felt numb or detached from people, activities, or surroundings?** Yes/No/does not want to answer<br>5. **Felt guilty or unable to stop blaming yourself or others for the pandemic or any problems the pandemic may have caused?** Yes/No/does not want to answer

Second questionnaire

2. General information about the participant

**What is your current professional situation? I will read you a list of options and I would like you to answer with the option that best describes your situation:**<br>- Works<br>- You are unemployed<br>- He is a student<br>- You are a working student<br>- You are retired<br>- Is engaged in unpaid domestic work (is a domestic worker or informal caregiver)<br>- You are permanently incapacitated (unable to work)<br>- Another<br>- You do not want to answer
***If the participant answered that he/she "Works", "Is a student" or "Is a working-student": Was there a change in the duration, in hours of work or school, compared to the situation he/she was in 3 months ago, at the time of the first questionnaire?**<br>- Yes<br>- No<br>- You do not want to answer
***If the answer to the previous question is <u>YES</u>: Was there an increase, decrease or did you need to stop?**<br>- Increase<br>- Decrease<br>- Need to stop<br>- Unknown<br>- You do not want to answer
***If the answer to the previous question is <u>DIMINING or NEEDING TO STOP</u>: What is the reason for the decrease or need to stop? I will read you a list of options and I would like you to respond with the option that best describes your situation or explain the reason:**<br>- Poor health;<br>- New carer status;<br>- Restrictions due to pandemic COVID-19;<br>- Outra ________________________

**What is your weight at today's date? If you do not know the exact figure, please answer an approximate value.**

____

"In this part of the survey, I would like you to answer some questions about your symptoms and your health today."

3. Symptoms and health of the participant today

***Question only if the participant is from the <u>POSITIVE TEST</u> group: For this question I will read you 5 alternatives and at the end I would like you to tell me which option you most identify with. Do you feel fully recovered from COVID-19?**<br>- Totally disagree<br>- I disagree<br>- I neither agree nor disagree<br>- I agree<br>- Totally agree<br>- You do not want to answer
**Now I am going to read you a list of various symptoms and I would like you to tell me whether or not, in the last 7 days, you have felt any or some of them, which you had not felt before the test was taken 9 months ago in August 2022. Some symptoms will be the same and some will be different from those I have already asked about. As you hear each symptom I would ask you to answer only yes, no or do not remember:**<br>38. **Persistent cough or worsening of usual cough** Yes/No/Don't know/would not answer<br>39. **Difficulty in breathing** Yes/No/Don't know/would not answer<br>40. **Runny or runny nose** Yes/No/Don't know/wouldn't answer<br>41. **Sore throat** Yes/No/Don't know/no answer<br>42. **Chest pain** Yes/No/Don't know/would not answer<br>43. **Belly ache** Yes/No/Don't know/wouldn't answer<br>44. **Vomiting or nausea** Yes/No/Don't know/would not answer<br>45. **Diarrhoea** Yes/No/Don't know/no answer<br>46. **Fever with temperature above 38°C on thermometer** Yes/No/Don't know/does not want to answer<br>47. **Chills** Yes/No/Don't know/No answer<br>48. **Headache** Yes/No/Don't know/no answer<br>49. **Joint pain** Yes/No/Don't know/no answer<br>50. **Muscle aches in the whole body** Yes/No/Don't know/wouldn't answer

51. **Change in smell** Yes/No/Don't know/no answer
52. **Taste change** Yes/No/Don't know/no answer
53. **Tiredness or lack of strength** Yes/No/Don't know/no answer
54. **Pain when breathing** Yes/No/Don't know/No answer
55. **Palpitations (or feeling your heart racing)** Yes/No/Don't know/wouldn't answer
56. **Loss of appetite** Yes/No/Don't know/no answer
57. **Difficulty in evacuating** Yes/No/Don't know/would not answer
58. **Difficulty in passing urine** Yes/No/Don't know/no answer
59. ***If MALE: Erectile dysfunction or unusual difficulties in achieving or maintaining an erection** Yes/No/Don't know/doesn't want to answer
60. ***If female and age <55 years: Changes in your usual menstrual cycle** Yes/No/Don't know/doesn't want to answer
61. **Swollen ankle(s)** Yes/No/Does not know/does not want to answer
62. **Balance problems** Yes/No/don't know/no answer
63. **Inability to feel one side of the body or face** Yes/No/Does not know/does not want to answer
64. **Tingling sensation** Yes/No/Don't know/would not answer
65. **Fainting episode** Yes/No/Don't know/would not answer
66. **Convulsions** Yes/No/Don't know/would not answer
67. **Tremors** Yes/No/Don't know/would not answer
68. **Difficulty in swallowing** Yes/No/don't know/no **answer**
69. **Difficulty in chewing** Yes/No/don't know/no answer
70. **Hearing a buzzing sound for most of a day** Yes/No/Don't know/doesn't want to answer
71. **Insomnia** Yes/No/Don't know/wouldn't answer
72. **Redness of the skin** Yes/No/Don't know/no answer
73. **Difficulty in concentrating** Yes/No/Does not know/does not want to answer
74. **Difficulty in remembering** Yes/No/Does not know/does not want to answer

***If you answered YES to the symptom Tiredness or lack of strength: On an intensity from 0 to 10, with 0 being no tiredness and 10 being extreme tiredness, how would you rate the intensity of your tiredness in the last 24 hours?**

____

***If you answered YES to at least one of the previous symptoms: Have you sought care for any of the symptoms you mentioned?**

- Yes
- No
- You do not want to answer

***If you answered <u>YES</u> to the previous question about seeking care: Have you been diagnosed with the post-COVID-19 condition?**<br>- Yes<br>- No<br>- You do not want to answer
***If you answered <u>YES to</u> at least one of the previous symptoms and answered that you WORK <u>or ARE WORK-STUDENT</u> in the question on work status: During the past seven days, to what extent have the symptoms you have experienced affected your productivity while at work? Please select a number from 0 to 10, where 0 is no effect on your work and 10 has completely prevented you from doing your work.**<br><br>____
***If you answered <u>YES to</u> at least one of the previous symptoms and answered that you WORK <u>or ARE WORK-STUDENT</u> in the question on work status: During the past seven days, to what extent have the symptoms you have experienced affected your ability to carry out your normal non-work related daily activities? Please select a number from 0 to 10, where 0 corresponds to no effect on daily activities and 10 has completely prevented you from carrying out your daily activities.**<br><br>____
**I am going to read you 4 options and I would like you to tell me which statement best describes your feeling of breathlessness TODAY:**<br>- I only experienced shortness of breath during intense physical exercise<br>- He would get short of breath when rushing or walking down a slightly sloping road<br>- He walked slower than other people because of shortness of breath, or had to stop for breath when walking at his normal pace<br>- I would stop for breath after walking 100 metres or after a few minutes<br>- felt too tired or out of breath to leave the house, get dressed or undressed<br>- You do not want to answer
**Now I will repeat the questions I asked you earlier about your state of health. We want to find out what you think about your health TODAY.**<br><br>**First I'd like to ask you about mobility. Would you say that:**<br>- You have no problems in walking about?<br>- You have slight problems in walking about?<br>- You have moderate problems in walking about?

- You have severe problems in walking about?
- You are unable to walk about?
- You do not want to answer

Next I'd like to ask you about self-care. Would you say that:

- You have no problems washing or dressing yourself?
- You have slight problems washing or dressing yourself?
- You have moderate problems washing or dressing yourself?
- You have severe problems washing or dressing yourself?
- You are unable to wash or dress yourself?
- You do not want to answer

Next I'd like to ask you about usual activities, for example work, study, housework, family or leisure activities. Would you say that:

- You have no problems doing your usual activities?
- You have slight problems doing your usual activities?
- You have moderate problems doing your usual activities?
- You have severe problems doing your usual activities?
- You are unable to do your usual activities?
- You do not want to answer

Next I'd like to ask you about pain or discomfort. Would you say that:

- You have no pain or discomfort?
- You have slight pain or discomfort?
- You have moderate pain or discomfort?
- You have severe pain or discomfort?
- You have extreme pain or discomfort?
- You do not want to answer

Finally I'd like to ask you about anxiety or depression. Would you say that:

- You are not anxious or depressed?
- You are slightly anxious or depressed?
- You are moderately anxious or depressed?
- You are severely anxious or depressed?
- You are extremely anxious or depressed?
- You do not want to answer

**Going back to the idea of the vertical line numbered from 0 to 100, with 100 being the best health you can imagine and 0 being the worst health you can imagine, I would ask you to tell me where on this scale you would place your health TODAY.**<br><br>____
**From the date of the first questionnaire until today, have you been diagnosed with any of these diseases? I will read out a list of diseases and would like you to answer Yes or No:**<br><br>10. **COVID-19** Yes/No/Don't know/wouldn't answer<br>1.1. **If <u>YES</u>: On what date were you diagnosed?** ____________<br>11. ***If you answered NO to question # from the first questionnaire: High Voltage** Yes/No/Don't know/wouldn't answer<br>12. ***If you answered NO to question # from the first questionnaire: Diabetes, excluding diabetes during pregnancy** Yes/No/Don't know/wouldn't answer<br>13. ***If you answered NO to question # from the first questionnaire: Asthma** Yes/No/Don't know/wouldn't answer<br>14. ***If you answered NO to question # from the first questionnaire: Chronic Bronchitis** Yes/No/Don't know/wouldn't answer<br>15. **Stroke (or heart attack)** Yes/No/Don't know/wouldn't answer<br>16. **Stroke** Yes/No/Don't know/would not answer<br>17. **Deep vein thrombosis (e.g. thrombosis on one leg)** Yes/No/Don't know/would not answer<br>18. **Pulmonary thromboembolism (thrombosis in the lung)** Yes/No/Don't know/would not answer<br><br>**Have you been diagnosed with any other illness that has not been mentioned?**<br>- Yes. **Which one?** ________________________<br>- No<br>- You do not want to answer

We will now enter the last phase of the questionnaire. I would like to ask you some questions about your emotional and psychological well-being. There are no right or wrong answers. We are only interested in your personal opinion.

5. Mental Health Scales

(PHQ - Depression Disorder Screening Scale)

During the last 14 days, on how many of these days have you been affected by any of the following problems?<br><br>**10. "I had little interest or pleasure in doing things":**<br>→ Never 0<br>→ On several days +1<br>→ On more than half the number of days +2<br>→ On almost every day +3<br>→ You do not want to answer<br><br>**11. "I felt discouraged, discouraged or hopeless"**<br>→ Never 0<br>→ On several days +1<br>→ On more than half the number of days +2<br>→ On almost every day +3<br>→ You do not want to answer
***If the total score for the last two statements is ≥3, apply the following questions:**<br><br>**12. "I had difficulty falling asleep or sleeping uninterruptedly, or I overslept"**<br>→ Never<br>→ On several days<br>→ On more than half the number of days<br>→ On almost every day<br>→ You do not want to answer<br><br>**13. "I felt tired or lacked energy"**<br>→ Never<br>→ On several days<br>→ On more than half the number of days<br>→ On almost every day<br>→ You do not want to answer<br><br>**14. "I had a lack or excess of appetite"**<br>→ Never<br>→ On several days<br>→ On more than half the number of days<br>→ On almost every day

→ You do not want to answer

15. "I felt that I don't like myself - or that I am a failure or that I have let myself or my family down"

→ Never
→ On several days
→ On more than half the number of days
→ On almost every day
→ You do not want to answer

16. "I found it difficult to concentrate on things, like when reading the newspaper or watching TV"

→ Never
→ On several days
→ On more than half the number of days
→ On almost every day
→ You do not want to answer

17. "I moved or spoke so slowly that other people might have noticed. Or the opposite: I was agitated to the point of moving around much more than usual.

→ Never
→ On several days
→ On more than half the number of days
→ On almost every day
→ You do not want to answer

18. "I thought it would be better to be dead, or to hurt myself in some way"

→ Never
→ On several days
→ On more than half the number of days
→ On almost every day
→ You do not want to answer

(GAD - Generalised Anxiety Disorder Screening Scale)

During the last 14 days, on how many days have you been affected by any of the following problems?

8. **"I felt nervous, anxious or irritable".**

→ Never 0
→ On several days +1
→ On more than half the number of days +2
→ On almost every day +3
→ You do not want to answer

9. **"I was unable to stop worrying or control the worries"**

→ Never 0
→ On several days +1
→ On more than half the number of days +2
→ On almost every day +3
→ You do not want to answer

***If the total score for the last two statements is ≥3, apply the following questions:**

10. **"I worried too much about different issues"**

→ Never
→ On several days
→ On more than half the number of days
→ On almost every day
→ You do not want to answer

11. **"I found it difficult to relax"**

→ Never
→ On several days
→ On more than half the number of days
→ On almost every day
→ You do not want to answer

12. **"I was so restless it was hard to be quiet**

→ Never
→ On several days

→ On more than half the number of days
→ On almost every day
→ You do not want to answer

13. "I was easily annoyed or irritable"
→ Never
→ On several days
→ On more than half the number of days
→ On almost every day
→ You do not want to answer

14. "I felt fear, as if something terrible might happen"
→ Never
→ On several days
→ On more than half the number of days
→ On almost every day
→ You do not want to answer

(PC-PTSD - Posttraumatic Stress Disorder Screening Scale)

To conclude the questionnaire, from an emotional and psychological point of view, I want to ask about your experience with the COVID-19 pandemic.

Sometimes events happen that are strangely or especially frightening, horrible or traumatic.

Do you consider the COVID-19 pandemic to be such an event?

- Yes
- No
- You do not want to answer

***If the answer to the previous question is YES, apply the following questions:**

In the past month, have you...

6. **Had nightmares about the pandemic or thought about the pandemic when you did not want to?** Yes/No/does not want to answer

7. **Tried hard not to think about the pandemic or went out of your way to avoid situations that reminded them of the pandemic?** Yes/No/does not want to answer
8. **Been constantly on guard, watchful, or easily startled?** Yes/No/does not want to answer
9. **Felt numb or detached from people, activities, or surroundings?** Yes/No/does not want to answer
10. **Felt guilty or unable to stop blaming yourself or others for the pandemic or any problems the pandemic may have caused?** Yes/No/does not want to answer
