# Supplementary material for: Long COVID is not the same for everyone: a hierarchical cluster analysis of Long COVID symptoms 9 and 12 months after SARS-CoV-2 test
Source: BMC Infect Dis. 2024 Sep 19;24:1001. doi: 10.1186/s12879-024-09896-8 (PMC11412022; doi:10.1186/s12879-024-09896-8)
Supplement: Supplementary file 3 — Supplementary Material 3. [file 12879_2024_9896_MOESM3_ESM.pdf]

Table 1 – Validation statistics for hierarchical clustering considering three and four clusters.

**Validation statistics**	**k=3**	**k=4**
n	552	552
cluster.number	3	4
min.cluster.size	22	22
noisen	0	0
average.between	2.38	2.27
average.within	1.49	1.42
max.diameter	4.90	4.90
min.separation	1	1
ave.within.cluster.ss	1.60	1.52
avg.silwidth	0.33	0.31
pearsongamma	0.52	0.55
dunn	0.20	0.20
dunn2	0.62	0.55
entropy	0.67	0.94
wb.ratio	0.63	0.63
ch	67.62	57.13
widestgap	3.32	3.32

Note: average.between - average distance between clusters; average.within - average distance within clusters; max.diameter - maximum cluster diameter; min.separation - minimum cluster separation; within.cluster.ss - a generalisation of the within clusters sum of squares - k-means objective function, which is obtained if d is a Euclidean distance matrix; avg.silwidth - average silhouette width; pearsongamma - correlation between distances and a 0-1 vector where 0 means same cluster, 1 means different clusters; dunn - Dunn index. Minimum separation / maximum diameter; dunn2 - minimum average dissimilarity between two cluster/maximum average within cluster dissimilarity - another version of the family of Dunn indexes; entropry - entropy of the distribution of cluster memberships; wb.ratio - average.within/average.between; widestgap - widest within-cluster gap.

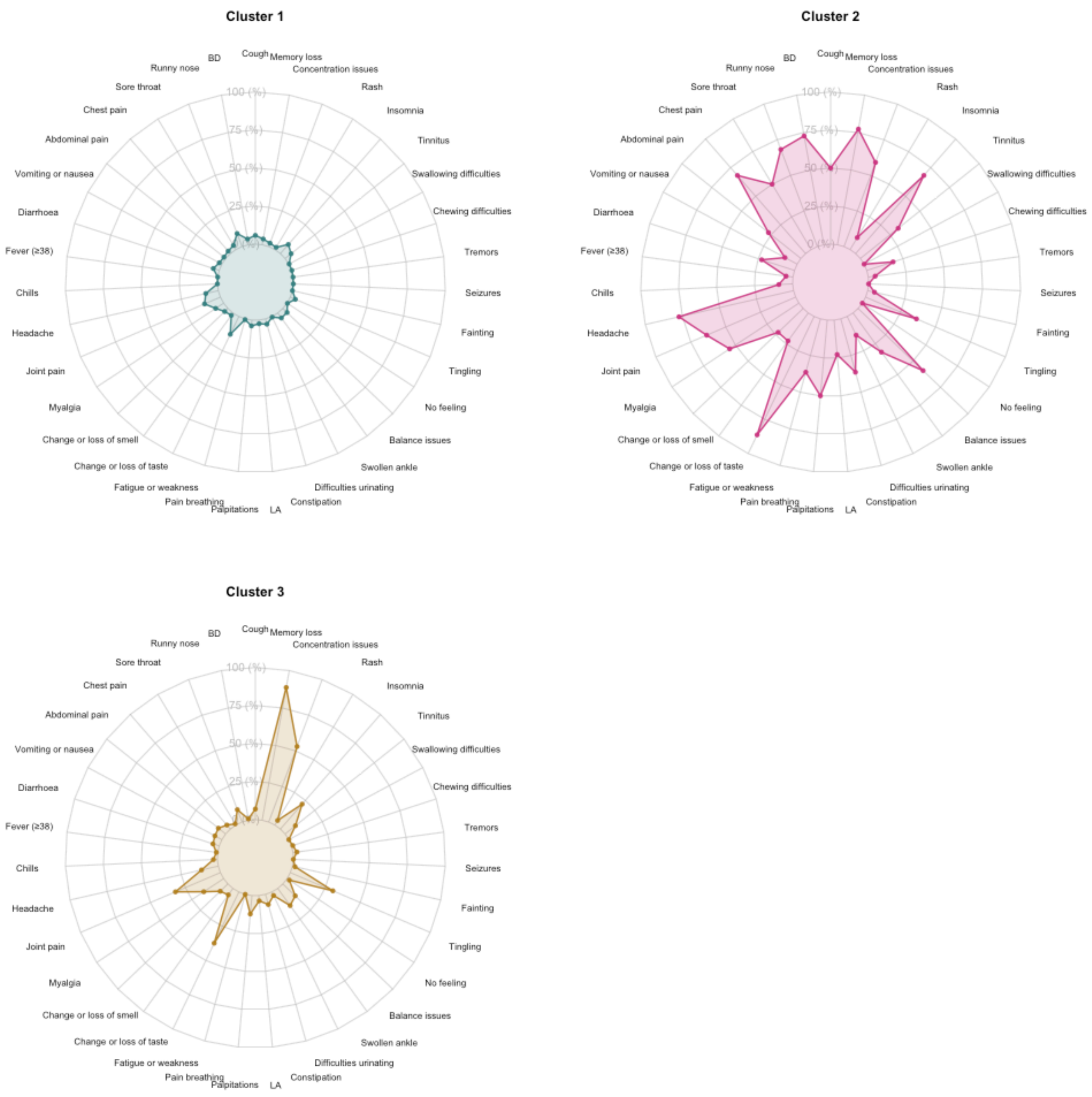

Figure 1 - Radar plots displaying the frequency of Long COVID symptoms across all four clusters. Abbreviations: LA: Loss of appetite; No feeling: Not feeling one side of the body or face; BD: Breathing difficulties.
